# Cerebrospinal fluid findings in patients with myelin oligodendrocyte glycoprotein (MOG) antibodies. Part 1: Results from 163 lumbar punctures in 100 adult patients

**DOI:** 10.1186/s12974-020-01824-2

**Published:** 2020-09-03

**Authors:** Sven Jarius, Hannah Pellkofer, Nadja Siebert, Mirjam Korporal-Kuhnke, Martin W. Hümmert, Marius Ringelstein, Paulus S. Rommer, Ilya Ayzenberg, Klemens Ruprecht, Luisa Klotz, Nasrin Asgari, Tobias Zrzavy, Romana Höftberger, Rafik Tobia, Mathias Buttmann, Kai Fechner, Kathrin Schanda, Martin Weber, Susanna Asseyer, Jürgen Haas, Christian Lechner, Ingo Kleiter, Orhan Aktas, Corinna Trebst, Kevin Rostasy, Markus Reindl, Tania Kümpfel, Friedemann Paul, Brigitte Wildemann, in cooperation with the Neuromyelitis Optica Study Group (NEMOS)

**Affiliations:** 1 Molecular Neuroimmunology Group, Department of Neurology, University of Heidelberg, Heidelberg, Germany; 2 Institute of Clinical Neuroimmunology, University Hospital and Biomedical Center, Ludwig-Maximilians University Munich, Munich, Germany; 3 Department of Neurology, Charité - Universitätsmedizin Berlin, Berlin, Germany; 4 Experimental and Clinical Research Center, Max Delbrueck Center for Molecular Medicine, and Charité Universitätsmedizin Berlin, Berlin, Germany; 5 Department of Neurology, Hannover Medical School, Hannover, Germany; 6 Department of Clinical Neuroimmunology and Neurochemistry, Hannover Medical School, Hannover, Germany; 7 Department of Neurology, Medical Faculty, Heinrich Heine University, Düsseldorf, Germany; 8 Department of Neurology, Center for Neurology and Neuropsychiatry, LVR-Klinikum, Heinrich Heine University, Düsseldorf, Germany; 9 Institute of Neurology, Medical University of Vienna, Vienna, Austria; 10 Department of Neurology, St Josef Hospital, Ruhr-University Bochum, Bochum, Germany; 11 Department of Neurology with Institute of Translational Neurology, University Hospital Münster, Münster, Germany; 12 Department of Regional Health Research, Odense, Denmark; 13 Department of Molecular Medicine, University of Southern Denmark, Odense, Denmark; 14 Department of Neuropathology, University of Göttingen, Göttingen, Germany; 15 Caritas Hospital Bad Mergentheim, Bad Mergentheim, Germany; 16 Euroimmun AG, Lübeck, Germany; 17 Department of Neurology, Medical University Innsbruck, Innsbruck, Austria; 18 Division of Pediatric Neurology, Department of Pediatrics I, Medical University of Innsbruck, Innsbruck, Austria; 19 Marianne-Strauß-Klinik, Behandlungszentrum Kempfenhausen für Multiple Sklerose Kranke gGmbH, Berg, Germany; 20 Department of Pediatric Neurology, Children’s Hospital Datteln, University Witten/Herdecke, Witten, Germany

**Keywords:** Myelin oligodendrocyte glycoprotein (MOG), Antibodies, Encephalomyelitis, Cerebrospinal fluid, Lumbar puncture, Optic neuritis, Transverse myelitis, Neuromyelitis optica (Devic syndrome), Acute disseminated encephalomyelitis (ADEM), Multiple sclerosis (MS), Oligoclonal bands, MOG antibody-associated disease (MOGAD)

## Abstract

**Background:**

New-generation cell-based assays have demonstrated a robust association of serum autoantibodies to full-length human myelin oligodendrocyte glycoprotein (MOG-IgG) with (mostly recurrent) optic neuritis, myelitis, and brainstem encephalitis, as well as with neuromyelitis optica (NMO)-like or acute-disseminated encephalomyelitis (ADEM)-like presentations. However, only limited data are yet available on cerebrospinal fluid (CSF) findings in MOG-IgG-associated encephalomyelitis (MOG-EM; also termed MOG antibody-associated disease, MOGAD).

**Objective:**

To describe systematically the CSF profile in MOG-EM.

**Material and methods:**

Cytological and biochemical findings (including white cell counts and differentiation; frequency and patterns of oligoclonal bands; IgG/IgM/IgA and albumin concentrations and CSF/serum ratios; intrathecal IgG/IgA/IgM fractions; locally produced IgG/IgM/IgA concentrations; immunoglobulin class patterns; IgG/IgA/IgM reibergrams; Link index; measles/rubella/zoster (MRZ) reaction; other anti-viral and anti-bacterial antibody indices; CSF total protein; CSF l-lactate) from 163 lumbar punctures in 100 adult patients of mainly Caucasian descent with MOG-EM were analyzed retrospectively.

**Results:**

Most strikingly, CSF-restricted oligoclonal IgG bands, a hallmark of multiple sclerosis (MS), were absent in almost 90% of samples (*N* = 151), and the MRZ reaction, the most specific laboratory marker of MS known so far, in 100% (*N* = 62). If present, intrathecal IgG (and, more rarely, IgM) synthesis was low, often transient and mostly restricted to acute attacks. CSF WCC was elevated in > 50% of samples (median 31 cells/μl; mostly lymphocytes and monocytes; > 100/μl in 12%). Neutrophils were present in > 40% of samples; activated lymphocytes were found less frequently and eosinophils and/or plasma cells only very rarely (< 4%). Blood–CSF barrier dysfunction (as indicated by an elevated albumin CSF/serum ratio) was present in 48% of all samples and at least once in 55% of all patients (*N* = 88) tested. The frequency and degree of CSF alterations were significantly higher in patients with acute myelitis than in patients with acute ON and varied strongly depending on attack severity. CSF l-lactate levels correlated significantly with the spinal cord lesion load in patients with acute myelitis (*p* < 0.0001). Like pleocytosis, blood–CSF barrier dysfunction was present also during remission in a substantial number of patients.

**Conclusion:**

MOG-IgG-positive EM is characterized by CSF features that are distinct from those in MS. Our findings are important for the differential diagnosis of MS and MOG-EM and add to the understanding of the immunopathogenesis of this newly described autoimmune disease.

## Introduction

Over the past few years, several studies using new-generation cell-based assays (CBA) have demonstrated a robust association of immunoglobulin G (IgG) autoantibodies targeting full-length, conformationally intact human myelin oligodendrocyte glycoprotein (MOG) with (mostly recurrent) optic neuritis (ON), myelitis, and brainstem encephalitis, as well as with neuromyelitis optica (NMO)-like and acute-disseminated encephalomyelitis (ADEM)-like presentations, rather than with classic multiple sclerosis (MS) [1–13]. Based on evidence from (a) immunological studies suggesting a direct pathogenic impact of MOG-IgG, (b) neuropathological studies demonstrating discrete histopathological features, (c) serological studies reporting a lack of aquaporin-4 (AQP4)-IgG in almost all MOG-IgG-positive patients, and (d) cohort studies suggesting differences in clinical and paraclinical presentation, treatment response and prognosis, MOG-IgG is now considered to denote a disease entity in its own right, distinct from classic MS and from AQP4-IgG-positive NMO spectrum disorders (NMOSD) [14–19], which is now often referred to as MOG-IgG-associated encephalomyelitis (MOG-EM) or MOG-IgG-associated autoimmune disease [11, 20, 21].

So far, only limited data are available on the cerebrospinal fluid (CSF) profile in MOG-EM. Previous studies were either based on relatively small patient numbers, included mainly pediatric patients, and/or did not take into account Caucasian patients. Moreover, all investigated only a small number of selected CSF parameters. Finally, none of the previous studies required confirmation of MOG-IgG seropositivity by means of a second assay. This is problematic, given the limited specificity of some of the currently available assays.

For this study, we systematically and comprehensively analyzed the results of 163 lumbar punctures (LP) from a cohort of 100 adult patients of mainly Caucasian descent with MOG-IgG-positive EM.

## Patients and methods

### Patients

Results from 163 lumbar punctures (LP) in 100 adult MOG-IgG-positive patients were analyzed retrospectively. MOG-EM was defined as monophasic or relapsing acute ON, myelitis, brainstem encephalitis, or encephalitis associated with MRI or (in the case of ON only) electrophysiological findings compatible with CNS demyelination and with MOG-IgG as detected by means of a cell-based assay (CBA) employing human full-length MOG as antigen [22]. Longitudinally extensive transverse myelitis (LETM) was defined as acute myelitis with at least one contiguous lesion extending over three or more vertebral segments (VS) as detected by T2-weighted or Gd-enhanced T1-weighted magnetic resonance imaging (MRI) [31, 32]. Cases of acute myelitis in which no lesion extended over more than two segments were classified as non-longitudinally extensive transverse myelitis (NETM). All patients were diagnosed with MOG-EM at German (Heidelberg, Berlin, Munich, Hanover, Düsseldorf, Bochum, Göttingen, Münster), Austrian (Vienna), and Danish (Odense) university hospitals. All eligible patients seen at the respective centers were included [22]. Assays used included three live CBA (Medical University Innsbruck, Austria; University of Vienna, Austria; Ludwig Maximilian University Munich, Germany) [1, 23–25], an in-house fixed CBA (University of Heidelberg, Germany) [2, 26] and a commercial fixed CBA (Euroimmun, Lübeck, Germany). Ninety-seven of 100 patients (97%) were tested by means of two or more independent CBA, including by at least one live CBA and one fixed CBA. None of the patients was positive for AQP4-IgG. Results from follow-up LP (i.e., any LP performed during the course of disease but after the first LP) were available for 36/100 (36%) patients. In total, 63 follow-up CSF examinations were performed (median 1 follow-up examination per patient; range 1-7). The first LP was performed after a median of 10 days after disease onset and the follow-up LP after a median of 16 days of the last previous attack; the proportion of samples taken during relapse did not significantly differ between the two groups (84.2% vs. 81.1%). The mean time interval between LPs was 424 days (median 79 days). The study was approved by the review boards of the participating centers. Patients gave written informed consent. LPs were performed for diagnostic purposes in all cases; no samples were obtained for this study.

### Evaluation of the humoral immune response

Oligoclonal IgG bands were assessed by isoelectric focusing and evaluated according to an international consensus [27]. Immunoglobulins and albumin were measured immunonephelometrically. Quantitative expressions of the intrathecal humoral immune response were based on the calculation of the CSF/serum quotients QIgG, QIgM, and QIgA with *Q*_Ig_ = Ig_CSF[mg/l]_/Ig_serum[g/l]_. The upper limits of the respective reference ranges, *Q*_lim_(IgG), *Q*_lim_(IgM), and *Q*_lim_(IgA), were calculated against QAlb according to Reiber’s revised hyperbolic function [28]. Values for Q_Ig_ exceeding *Q*_lim_(Ig) were considered to indicate intrathecal immunoglobulin synthesis [28]. The fraction (in %) of intrathecally produced Ig (Ig_IF_) and the absolute amount of locally, i.e., intrathecally, produced Ig (IgG_loc_) were calculated according to the following formulas: Ig_IF[%]_ = [*Q*IgG − *Q*_lim_(Ig)] × Ig_serum_ × 100 and Ig_loc[mg/L]_ = [*Q*_Ig_ − *Q*_lim_(Ig)] × Ig_serum_, respectively [28]. CSF and serum concentrations for immunoglobulins and albumin, respectively, were analyzed within the same analytical series.

### Evaluation of the blood–CSF barrier

The CSF/serum albumin quotient, QAlb = Alb_CSF[mg/l]_/Alb_serum[g/l]_, was used to assess the blood–CSF barrier (BCB) function. As the upper reference limit of QAlb is age-dependent, *Q*_lim_(Alb) was calculated as 4 + (a/15) × 10^−3^ with a representing patient’s age according to Reiber et al. (1994) [29]. Dysfunction of the BCB was defined as QAlb > *Q*_lim_(Alb).

### Cytological examination, total CSF protein, and l-lactate

A white cell count > 5/μl was classified as increased [30]. An age-dependent reference range for CSF l-lactate was applied (16–50: 2.1 mmol/l, > 50: 2.6 mmol/l) [30]. The upper reference limit for total CSF protein was set at 0.45 mg/l [30].

### Statistics

Samples were analyzed in total as well as after stratification according to disease status and treatment status. Fisher’s exact test, the Mann*–*Whitney *U* test, and the Kruskal*–*Wallis test were used to detect statistical differences between groups. Spearman’s rho was assessed to test for correlations. Due to the exploratory nature of this study, no correction for multiple testing was applied other than Dunn’s post test. Reiber diagrams (reibergrams) were generated using *Protein Statistics in CSF analysis V3.0* software (Comed, Soest, Germany).

## Results

### Patient characteristics

The median age at LP was 38 years (range 18-78). The male to female ratio was 1:1.4. A total of 92.6% of all samples were obtained from patients of Caucasian descent; 7.4% from patients of either Arabian or Asian descent. The median disease duration was ≤ 1 month at the time of LP (maximum 489 months) and 29 months (range 0-511) at the last follow-up. Information on the date of onset of the last attack prior to LP was available from the patient records for 148 samples. Of those, 123 (83.1%) were obtained within 45 days (median 9 days; range 0-44) after the onset of an acute attack (acute myelitis with or without other symptoms in 45.5% [“acute MY subgroup”]; acute ON but no myelitis in 43.1% [“acute ON subgroup”]; neither myelitis nor ON but isolated brain or brainstem/cerebellar disease in 8.9% [“acute BRAIN subgroup”]; missing data for 3 samples). Twenty-five samples were obtained more than 90 days after attack onset (“remission subgroup”). Thirty-five (63.6%) of 55 samples from patients with acute myelitis and available MRI data were obtained during episodes of LETM. The median cumulative spinal cord lesion load (summing up lesions in patients with multiple lesions) was 5 VS (up to 21 VS) in the total myelitis group and 6 VS (range 3-21) in the LETM subgroup. Of the ON samples, 71.7% were taken during attacks of unilateral and 22.6% during attacks of bilateral ON. Attack severity was classified by the treating physicians as “mild” or “moderate” in 64.9% and as “severe” in 27.2% (no data in the remainder). At last follow-up, 77% of all patients had experienced at least two attacks (“relapsing subgroup”) and 23% of patients had not relapsed (“monophasic subgroup”). At first LP, 67/98 (68.4%) patients were neither treated with steroids nor with immunosuppressants or immunomodulatory drugs at the time of LP (no precise data on the treatment status at the time of LP available for 2 patients). If all LPs are considered, 98/163 (60.1%) were obtained from patients who were untreated at the time of LP (no precise data for 5 samples).

### Cellular immune response

An increased CSF white cell count (WCC) was found in 82/159 (51.6%) samples examined, with a median of 31 cells/μl (range, 6-463). WCC ≥ 50 cells/μl, which are very rare in MS (and thus considered a 'red flag' that should prompt physicians to challenge a diagnosis of MS) [27, 33], were present in 19.1% (30/157) of samples. Severe pleocytosis, defined as WCC ≥ 100 cells/μl, was found in 19/157 (12.1%) samples (median, 177; range, 108-463), 18 of which were taken during an acute attack (unknown disease activity in the remaining patient) and 13 of which were obtained from untreated patients. CSF WCC exceeded 200 cells/μl only in 7/157 (4.5%) samples and 300 cells/μl in 3 samples (1.9%). In total, pleocytosis was noted at least once in 56/99 (56.6%) patients with available data.

Lymphocytes, found in 77/77 (100%) samples with available cytological data, and monocytes, detected in 57/77 (74%) samples, were the predominant immune cell types in the CSF. Relative lymphocyte counts ranged between 33% and 100% (median 80%; *N* = 53) of all CSF cells and relative monocyte counts between 1% and 75% (median 20%; *N* = 47).

Importantly, however, neutrophils were present in at least 42.9% (33/77) of samples. Neutrophils represented up to 66% of all leukocytes (data available for 29 samples) and up to 41% in samples with pleocytosis (*N* = 19). If only LPs with pleocytosis and available cytological data are considered, neutrophil granulocytes were present even in 50% (22/44) of samples. Neutrophils were more commonly found during acute attacks in the MY and BRAIN subgroups (57% of all samples with available data) than in the acute ON subgroup (25%). In total, neutrophil granulocytes were present at least once in 26/56 (46.4%) patients with available cytology data.

By contrast, eosinophils and basophils were rare findings, present in only 2/77 (2.6%) and 2/77 (2.6%) samples, respectively. Activated lymphocytes were noted in 12/77 (15.6%) samples and plasma cells in 3/77 (3.9%).

**Fig. 1:**
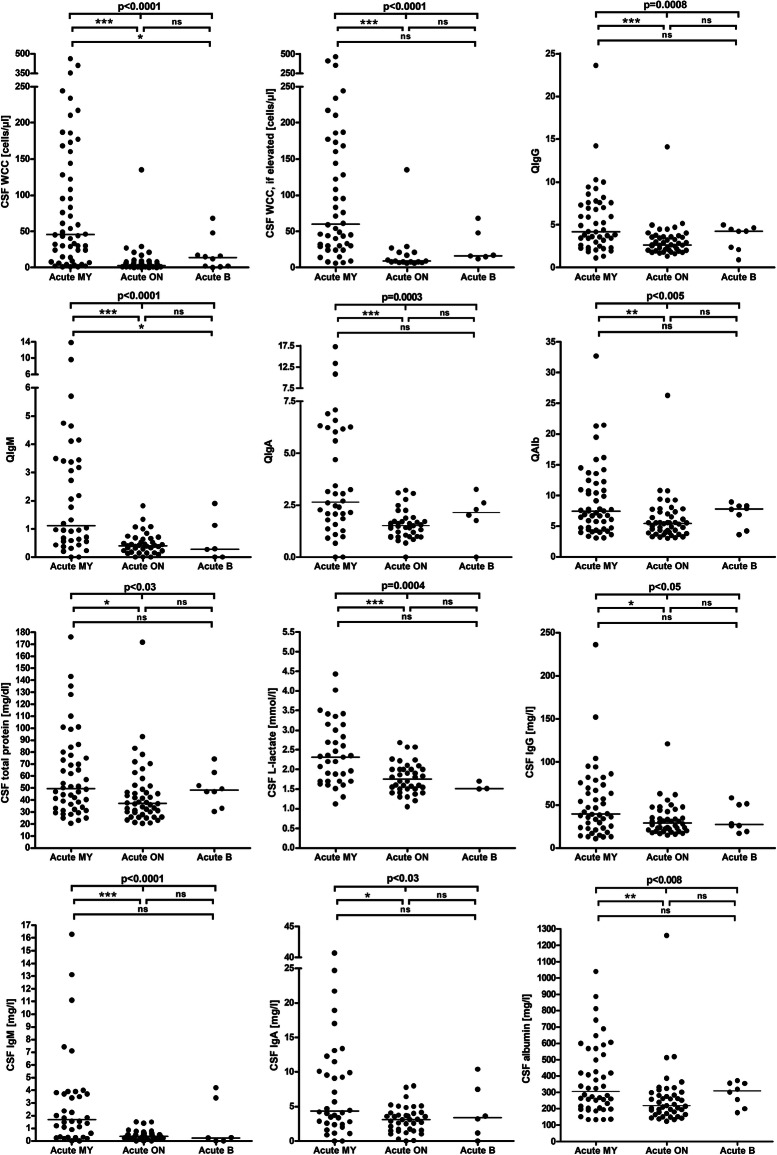
CSF white cell counts, IgG, IgA, IgM, and albumin CSF/serum ratios and CSF concentrations, CSF total protein concentrations, and CSF L-lactate concentrations in MOG-IgG-positive EM. A statistically significant difference between the acute MY subgroup and the acute ON subgroup was found with regard to all parameters studied. IgG/A/M, immunoglobulin G/A/M; QIgG/A/M, CSF/serum IgG/A/M ratios; QAlb, CSF/serum albumin ratio

Pleocytosis was significantly less common in the acute ON subgroup than in the acute MY subgroup (34% vs. 85.2%) (*p* < 0.000001). Similarly, median cell numbers were lower in the acute ON subgroup (3, range 0-135, vs. 45.5 cells/μl, range, 1-463) (*p* < 0.000001) (Fig. 1); CSF WCC ≥ 50 cells/μl were found almost exclusively in patients with acute myelitis at the time of LP (present in 46.3%, or 25/54, vs. 1.9%, or 1/52, in the acute ON subgroup). See Table 1 and Supplementary Figure 1 for further details.

**Table 1:** CSF white cell counts (WCC) and cytology results in MOG-IgG-positive EM

	Units	Total	Attack	Remission	Acute MY subgroup	Acute ON subgroup	Acute BRAIN subgroup
CSF white cell counts							
Pleocytosis	*Samples*	82/159 (51.6%)	72/120 (60%)	7/25 (28%)	46/54 (85.2%)	18/53 (34%)	6/10 (60%)
WCC, all samples	*Cells/μl*	6 (0-463; 155)	9 (0-463; 118)	2.5 (0-37; 24)	45.5 (1-463; 54)	3 (0-135; 51)	13.5 (0-68; 10)
WCC, if elevated	*Cells/μl*	31 (6-463; 80)	40 (6-463; 71)	21 (13-37; 7)	60.15 (6-463; 46)	9 (6-135; 17)	16.5 (12-68; 6)
WCC, > = 100	*Samples*	19/157 (12.1%)	18/119 (15.1%)	0/25 (0%)	17/54 (31.5%)	1/52 (1.9%)	0/10 (0%)
WCC, > = 100	*Cells/μl*	177 (108-463; 19)	181.5 (108-463; 18)	n.a. (n.a.; 0)	186 (108-463; 17)	135 (135-135; 1)	n.a. (n.a.; 0)
Lymphocytes	*Samples*	77/77 (100%)	58/58 (100%)	14/14 (100%)	30/30 (100%)	24/24 (100%)	4/4 (100%)
Monocytes	*Samples*	57/77 (74%)	42/58 (72.4%)	11/14 (78.6%)	20/30 (66.7%)	18/24 (75%)	4/4 (100%)
Neutrophils	*Samples*	33/77 (42.9%)	26/58 (44.8%)	6/14 (42.9%)	17/30 (56.7%)	6/24 (25%)	3/4 (75%)
Eosinophils	*Samples*	2/77 (2.6%)	2/58 (3.4%)	0/14 (0%)	1/30 (3.3%)	0/24 (0%)	1/4 (25%)
Basophils	*Samples*	2/77 (2.6%)	2/58 (3.4%)	0/14 (0%)	2/30 (6.7%)	0/24 (0%)	0/4 (0%)
Plasma cells	*Samples*	3/77 (3.9%)	2/58 (3.4%)	1/14 (7.1%)	2/30 (6.7%)	0/24 (0%)	0/4 (0%)
Lymphoid cells	*Samples*	12/77 (15.6%)	10/58 (17.2%)	0/14 (0%)	6/30 (20%)	4/24 (16.7%)	0/4 (0%)
Macrophages	*Samples*	6/77 (7.8%)	3/58 (5.2%)	2/14 (14.3%)	0/30 (0%)	2/24 (8.3%)	1/4 (25%)
No pleocytosis	*Samples*	77/159 (48.4%)	48/120 (40%)	18/25 (72%)	8/54 (14.8%)	35/53 (66%)	4/10 (40%)

WCC in the various subgroups are reported as medians; ranges and total sample numbers are given in brackets

**Fig. 2:**
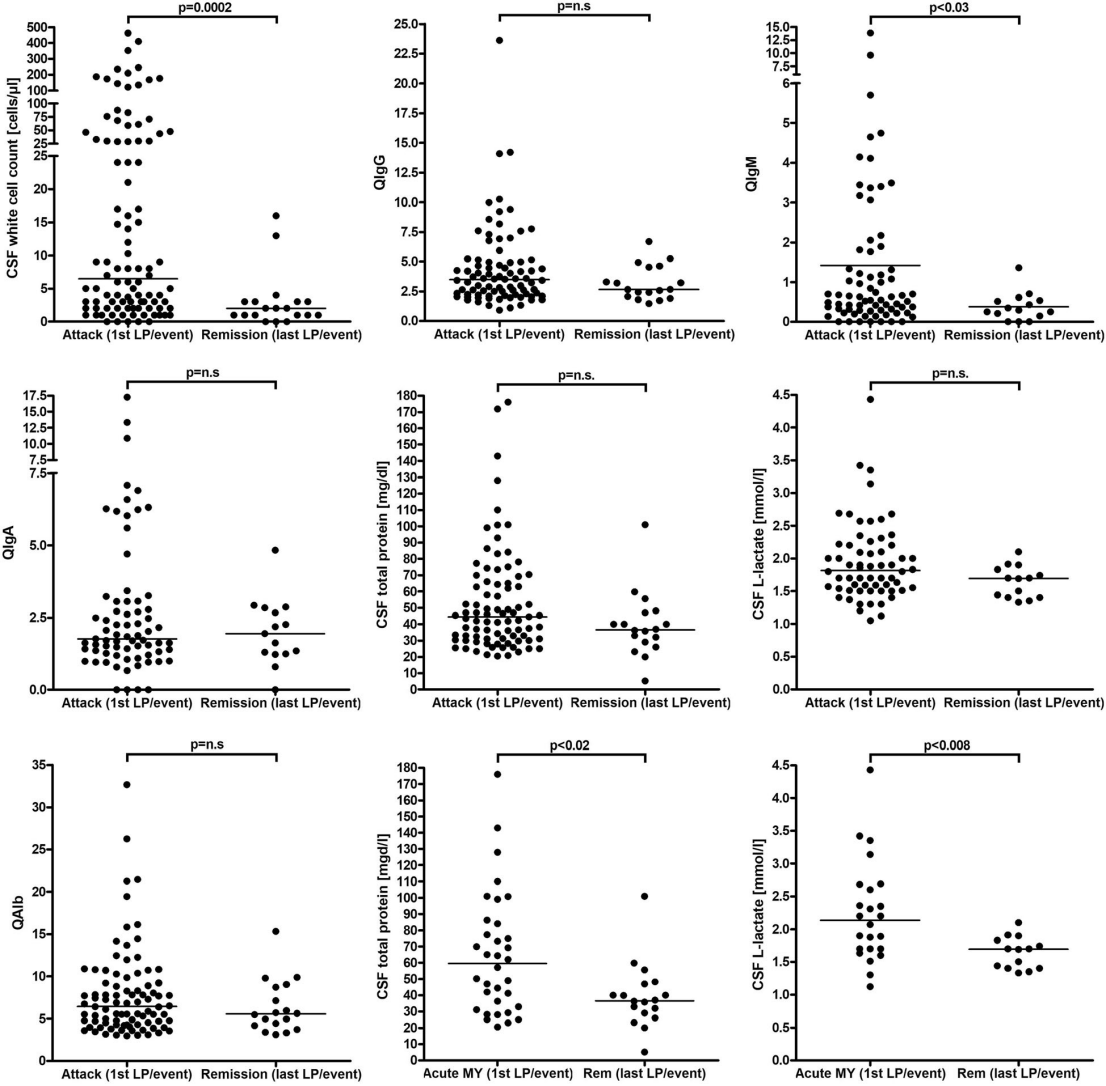
CSF white cell counts, IgG, IgA, IgM, and albumin CSF/serum ratios and CSF concentrations, CSF total protein concentrations, and CSF L-lactate concentrations during acute attacks and remission in MOG-IgG-positive EM. IgG/A/M, immunoglobulin G/A/M; MY, myelitis; QIgG/A/M, CSF/serum IgG/A/M ratios; QAlb, CSF/serum albumin ratio

Pleocytosis was also significantly more frequent in samples obtained during acute attacks (*p* < 0.005); similarly, cell numbers were significantly higher during acute attacks (*p* < 0.0007) (Fig. 2, Table 1, and Supplementary Figure 1). While activated lymphocytes, eosinophils, and basophils were noted only during acute attacks, neutrophils were found with similar frequency during acute attacks and in remission (44.8% vs. 42.9%).

**Fig. 3:**
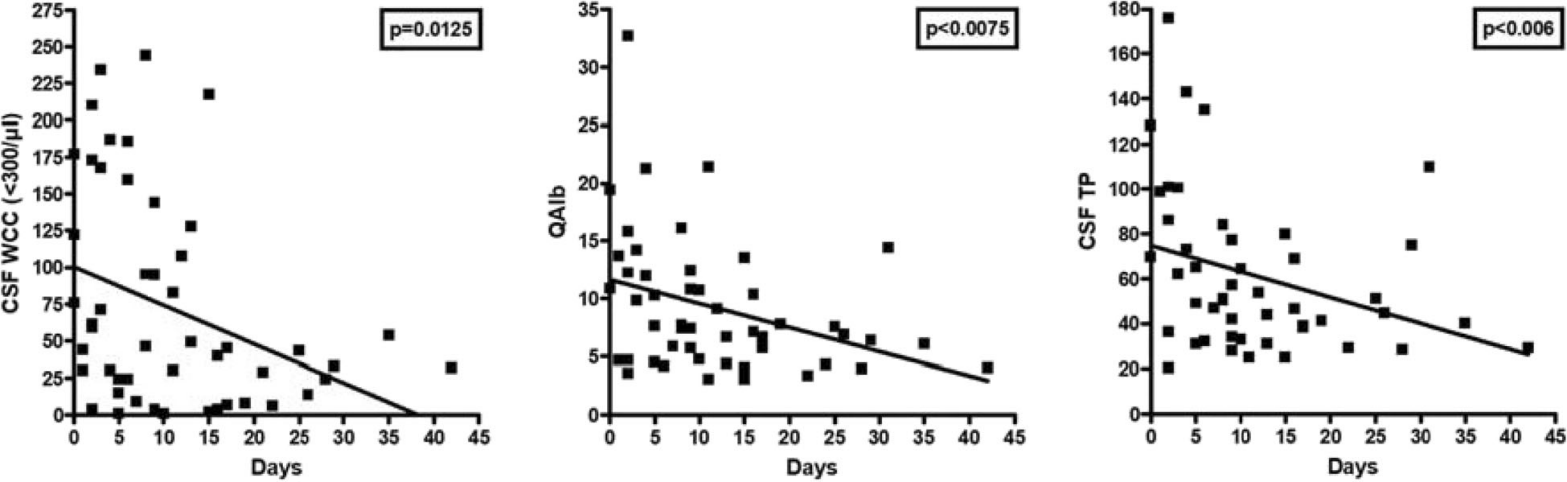
Correlation analyses for CSF white cell counts (after exclusion of three outliers >300 cells/μl), QAlb and CSF total protein, respectively, with time since attack onset in patients with acute MOG-IgG-positive myelitis

**Fig. 4:**
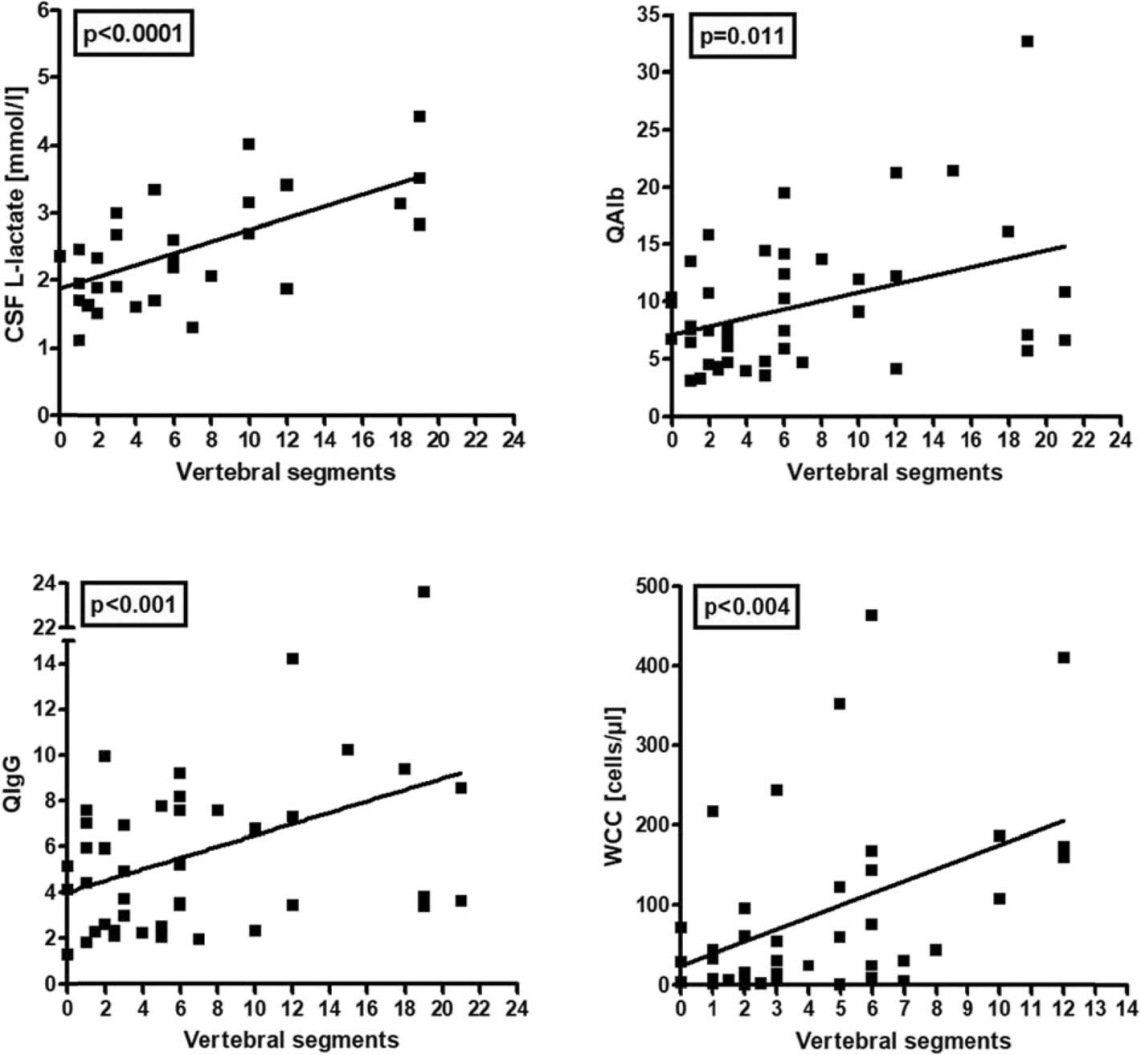
Correlation of CSF l-lactate concentrations, QAlb, QIgG, and CSF WCC with the spinal cord lesion load (as measured in vertebral segments) in patients with acute MOG-IgG-positive myelitis. Note that the CSF WCC correlated with the spinal lesion load only after exclusion of outliers with extraordinarily high lesion load (15 or more segments). The most significant correlation was found with CSF l-lactate levels. QAlb, albumin CSF/serum ratio, QIgG, IgG CSF/serum ratio; WCC, white cell count

After exclusion of three outliers with excessive WCC (≥ 300/μl), a significant negative correlation of CSF WCC with days since attack onset was noted in patients with acute myelitis (*r* = − 0.347, *p* < 0.02) (Fig. 3). No significant correlation between WCC and the cumulative spinal cord lesion load during acute myelitis attacks could be found in the total cohort, but WCC were correlated with spinal cord lesion load after exclusion of outliers with unusually high lesion load (≥ 15 VS) (*r*=0.461, *p* < 0.004) (Fig. 4).

### Intrathecal IgG synthesis

CSF-restricted OCB were positive in only 20/151 (13.2%) samples (OCB pattern 2 in 14/150 or 9.3%; pattern 3 in 5/150 or 3.3%; no data in 1), and QIgG was elevated in just 11/133 (8%) (median QIgG 4; range 2.3-7) (Table 2). In 28/150 (18.7%) samples, identical OCB in serum and CSF but no CSF-restricted bands were present (pattern 4). Pattern 5, indicating monoclonal gammopathy, was present in 2/150 samples (1.3%) from two patients. Overall, 15/100 (15%) patients showed CSF-restricted OCB at least once, and QIgG was positive in 9/88 (10.2%) patients at least once.

**Table 2:** Frequency of intrathecal IgG synthesis, oligoclonal IgG pattern, IgG CSF/serum ratios, intrathecal IgG fractions, absolute amount of locally produced IgG, and absolute IgG concentrations in the CSF and serum

	Units	Total	Attack	Remission	Acute MY subgroup	Acute ON subgroup	Acute BRAIN subgroup
Intrathecal IgG synthesis							
OCB positive or IgG-IF ≥ 10%	*Samples*	20/151 (13.2%)	13/112 (11.6%)	2/24 (8.3%)	7/50 (14%)	3/51 (5.9%)	2/10 (20%)
OCB positive	*Samples*	20/151 (13.2%)	13/112 (11.6%)	2/24 (8.3%)	7/50 (14%)	3/50 (6%)	2/10 (20%)
OCB pattern 1	*Samples*	101/150 (67.3%)	77/112 (68.8%)	18/24 (75%)	30/50 (60%)	41/50 (82%)	5/10 (50%)
OCB pattern 2	*Samples*	14/150 (9.3%)	9/112 (8%)	2/24 (8.3%)	3/50 (6%)	3/50 (6%)	2/10 (20%)
OCB pattern 3	*Samples*	5/150 (3.3%)	4/112 (3.6%)	0/24 (0%)	4/50 (8%)	0/50 (0%)	0/10 (0%)
OCB pattern 4	*Samples*	28/150 (18.7%)	20/112 (17.9%)	4/24 (16.7%)	11/50 (22%)	6/50 (12%)	3/10 (30%)
OCB pattern 5	*Samples*	2/150 (1.3%)	2/112 (1.8%)	0/24 (0%)	2/50 (4%)	0/50 (0%)	0/10 (0%)
OCB pattern 2 or 3	*Samples*	19/150 (12.7%)	13/112 (11.6%)	2/24 (8.3%)	7/50 (14%)	3/50 (6%)	2/10 (20%)
OCB pattern 3 or 4	*Samples*	33/150 (22%)	24/112 (21.4%)	4/24 (16.7%)	15/50 (30%)	6/50 (12%)	3/10 (30%)
OCB pattern 1, 4, or 5	*Samples*	131/150 (87.3%)	99/112 (88.4%)	22/24 (91.7%)	13/50 (86%)	6/50 (94%)	3/10 (80%)
QIgG > Q_lim_(IgG)	*Samples*	11/133 (8%)	9/100 (9%)	1/23 (4%)	6/47 (12.8%)	2/44 (4.5%)	1/8 (12.5%)
QIgG, all LPs	*-*	3.5 (0.9-23.6; 131)	3.5 (0.9-23.6; 99)	3.2 (1.5-14.7; 23)	4.2 (1.11-23.61; 47)	2.66 (1.32-14.09; 43)	4.24 (0.9-4.96; 8)
QIgG, if positive	*-*	4 (2.3-7; 10)	4.5 (2.37; 9)	3.2 (3.2-3.2; 1)	5.92 (2.26-7.01; 6)	4.02 (3.57-4.47; 2)	2.37 (2.37-2.37; 1)
IgG IF, all LPs	*% IgG(CSF)*	0 (0-53.3; 131)	0 (0-53.3; 99)	0 (0-6.8; 23)	0 (0-53.3; 47)	0 (0-26.4; 43)	0 (0-1.1; 8)
IgG IF, QIgG positives	*% IgG(CSF)*	10.5 (1.13-53.3; 10)	14.2 (1.1-53.3; 9)	6.8 (6.8-6.8; 1)	11.9 (4.5-53.3; 6)	20.3 (14.2-26.4; 2)	1.1 (1.1-1.1; 1)
IgG IF, > 10%	*Samples*	5/131 (3.8%)	5/99 (5.1%)	0/23 (0%)	3/47 (6.4%)	2/43 (4.7%)	0/8 (0%)
IgG Loc, all LPs	*mg/l*	0 (0-25; 127)	0 (0-25; 96)	0 (0-2.1; 22)	0 (0-25; 45)	0 (0-0.3; 8)	0 (0-25; 96)
IgG Loc, QIgG positives	*mg/l*	2.3 (0.29-25; 9)	4.7 (0.3-25; 8)	2.1 (2.1-2.1; 1)	2.3 (1.1-25; 5)	9.7 (7.9-11.6; 2)	0.3 (0.3-0.3; 1)
IgG CSF, all LPs	*mg/l*	33.1 (11-236.1; 129)	33.3 (11-236.1; 96)	32.4 (13-182; 22)	39.8 (11-236.1; 45)	28.9 (14.6-121; 42)	27 (17-58; 8)
IgG CSF, QIgG positives	*mg/l*	38 (22.5-75.7; 9)	41 (22.5-75.7; 8)	30.9 (30.9-30.9; 1)	38 (22.5-75.7; 5)	49.7 (44-55.4; 2)	25.8 (25.8-25.8; 1)
IgG serum, all LPs	*g/l*	9.9 (4-31.8; 127)	9.9 (4-31.8; 96)	9.8 (6-12.4; 22)	9.4 (4-31.8; 45)	10.2 (5.86-23.9; 42)	10.84 (4-31; 8)
IgG serum, QIgG positives	*g/l*	10 (6.3-15.5; 9)	10.4 (6.3-15.5; 8)	9.6 (9.6-9.6; 1)	10 (6.3-11.1; 5)	12.7 (9.8-15.5; 2)	10.9 (10.9-10.9; 1)
Link index, all	*Samples*	13/131 (10%)	9/99 (9%)	4/23 (17%)	7/47 (14.9%)	2/43 (4.7%)	0/8 (0%)
Link index, if positive	*Index*	0.8 (0.7-1.7; 13)	0.8 (0.7-1.5; 9)	1.2 (0.7-1.7; 4)	0.8 (0.7-1.5; 7)	0.9 (0.8-0.9; 2)	n.a. (n.a.; 0)

Quotients, indices, concentrations, and fractions are given as median and range

*QIgG/A/M* CSF/serum IgG/A/M ratio, *IgG/A/M IF* intrathecally produced IgG/IgA/IgM fraction, *IgG/A/M loc* locally (intrathecally) produced IgG/A/M, *LP* lumbar puncture

Not only the frequency but also the degree of intrathecal synthesis (IS) was low: QIgG was elevated in only 41.2% of the OCB-positive samples. In those samples with elevated QIgG, the intrathecal IgG fraction exceeded the first decile (values < 10% may result from the imprecision of nephelometric IgG testing and should thus not be taken as strict proof of IS according to current guidelines [30]) in only 19% of cases, the median IF-IgG was just 10.5% (range 1.13-53.3%), and the median absolute amount of intrathecally produced IgG was just 2.3 mg/l (range 0.29 to 25 mg/l).

While OCB were detectable with similar frequency both during acute attacks (13/112; 11.6%) and in remission (2/24; 8.3%), larger amounts of intrathecally produced IgG as indicated by an IgG-IF > 10% were found exclusively in samples obtained during acute attacks (Table 2). To evaluate whether the frequency of OCB increases with disease duration, we compared samples obtained within the first month since disease onset and samples obtained more than 1 year after disease onset but found no statistically significant difference regarding the rate of OCB positive samples (15.9% or 10/63, vs. 6.7% or 3/45); if only samples taken during acute attacks are considered, no sample obtained > 1 year after disease onset was positive for OCB (15.9% or 10/63, vs. 0% or 0/27; *p* < 0.03).

CSF IgG concentrations exceeded the reference range of 40 mg/l in 37/111 (33.3%) OCB-negative samples. However, in none of these cases QIgG was elevated. This indicates that the high CSF IgG concentrations in these samples were caused by passive transfer of IgG rather than by intrathecal synthesis. In fact, QAlb was increased in 94% (34/36) of these patients, indicating blood CSF barrier dysfunction.

There was a trend towards a higher frequency of CSF-restricted OCB (pattern 2 or 3) in the acute MY subgroup than in the acute ON subgroup (14% vs. 6%; *p* = n.s.) (Table 2). Similarly, QIgG was slightly more frequently elevated (12.8% vs. 4.5%; *p* = n.s.) and median IgG CSF/serum ratios (4.2 vs. 2.66; *p* < 0.0002) as well as CSF IgG levels (39.8 vs. 28.9 mg/dl; *p* < 0.02) were significantly higher in the acute MY subgroup (Table 2 and Fig. 1). In patients with acute myelitis, QIgG also correlated with the spinal cord lesion load (as measured in VS) (*r* = 0.391, *p* < 0.01) (Fig. 4).

IgG serum levels were elevated in 4/127 (3.1%) samples, all of which were taken during acute attacks. Median IgG serum concentrations did not differ significantly between acute samples and samples obtained during remission (Table 2) and also not between the acute MY and the acute ON subgroup (Supplementary Figure 2).

### Intrathecal IgM synthesis

QIgM was increased in 13/112 (12%) samples (median 1.9; range, 0.6-9.6) from 10 patients. In those samples with elevated QIgM, the fraction of intrathecally produced IgM varied between 1 and 59.1% (median 9.2%), corresponding to a median absolute amount of intrathecally produced IgM of 0.32 mg/l (range 0.01-2.3), and was > 10% only in 6 out of those 13 samples. As with IgG, an intrathecal IgM fraction of > 10% was observed only during acute attacks. Also QIgM was exclusively elevated during acute attacks (Table 3). More samples in the acute MY subgroup than in the acute ON subgroup showed IgM IS (24% vs. 8%), but the difference was not statistically significant. Median QIgM and the median CSF IgM concentration were significantly higher in the MY subgroup than in the ON subgroup (*p* < 0.000007 and *p* < 0.008, respectively) (Fig. 1).

**Table 3:** Frequency of intrathecal IgM and IgA synthesis, IgM and IgA CSF/serum ratios, intrathecal IgM and IgA fractions, amount of locally produced IgM and IgA, and absolute IgM and IgA concentrations in the CSF and serum

	Units	Total	Attack	Remission	Acute MY subgroup	Acute ON subgroup	Acute BRAIN subgroup
Intrathecal IgM synthesis							
QIgM > Q_lim_(IgM)	*Samples*	13/112 (12%)	13/84 (15%)	0/20 (0%)	9/38 (24%)	3/39 (8%)	1/6 (17%)
QIgM, all LPs	*-*	0.5 (0-13.8; 111)	0.6 (0-13.8; 83)	0.5 (0-4.1; 20)	1.11 (0-13.81; 38)	0.41 (0-1.82; 38)	0.28 (0-1.9; 6)
QIgM, if positive	*-*	1.9 (0.6-9.6; 13)	1.9 (0.6-9.6; 13)	n.a.	2.72 (0.63-9.63; 9)	0.84 (0.68-1.33; 3)	1.9 (1.9-1.9; 1)
IgM IF, all LPs	*% IgM* (*CSF*)	0 (0-59.1; 111)	0 (0-59.1; 83)	0 (0-0; 20)	0 (0-59.1; 38)	0 (0-41.9; 38)	0 (0-18.3; 6)
IgM IF, QIgM positives	*% IgM* (*CSF*)	9.2 (1-59.1; 13)	9.2 (1-59.1; 13)	n.a. (n.a.;0)	9.2 (3.5-59.1; 9)	9 (1-41.9; 3)	18.3 (18.3-18.3; 1)
IgM IF, > 10%	*Samples*	6/111 (5.4%)	6/83 (7.2%)	0/20 (0%)	4/38 (10.5%)	1/38 (2.6%)	1/6 (16.7%)
IgM Loc, all LPs	*mg/l*	0 (0-2.3; 108)	0 (0-2.3; 81)	0 (0-0; 19)	0 (0-2.3; 36)	0 (0-0.3; 38)	0 (0-0.6;6)
IgM Loc, QIgM positives	*mg/l*	0.32 (0.01-2.3; 13)	0.32 (0.01-2.3; 13)	n.a.	0.3 (0.1-2.3; 9)	0 (0-0.3; 3)	0.6 (0.6-0.6; 1)
IgM CSF	*mg/l*	0.5 (0-16.3; 110)	0.5 (0-16.3; 82)	0.49 (0-3.6; 19)	1.69 (0-16.3; 36)	0.36 (0-1.51; 39)	0.25 (0-4.19; 6)
IgM serum	*g/l*	1.02 (0.15-3.73; 112)	1.03 (0.15-3.73; 84)	0.89 (0.39-2.19; 20)	1.18 (0.3-2.96; 37)	0.94 (0.15-2.41; 40)	1 (0.79-3.73; 6)
Intrathecal IgA synthesis							
QIgA > Q_lim_(IgA)	*Samples*	5/111 (5%)	4/83 (5%)	1/20 (5%)	2/38 (5.3%)	2/38 (5.3%)	0/6 (0%)
QIgA, all LPs	*-*	2 (0-17.3; 110)	1.9 (0-17.3; 82)	2.5 (0-11.1; 20)	2.65 (0-17.27; 38)	1.51 (0-3.22; 37)	2.15 (0-3.26; 6)
QIgA, if positive	*-*	2.6 (1.7-6.2; 5)	2.4 (1.7-6.2; 4)	2.9 (2.9-2.9; 1)	4.42 (2.61-6.24; 2)	1.96 (1.68_2.24; 2)	n.a. (n.a.; 0)
IgA IF, all LPs	*% IgA* (*CSF*)	0 (0-34.2; 110)	0 (0-31.7; 82)	0 (0-34.2; 20)	0 (0-31.7; 38)	0 (0-13.3; 37)	0 (0-0; 6)
IgA IF, QIgA positives	*% IgA* (*CSF*)	13.3 (3.8-34.2; 5)	9.5 (3.8-31.7; 4)	34.2 (34.2-34.2; 1)	17.8 (3.8-31.7; 2)	9.5 (5.7-13.3; 2)	n.a. (n.a.; 0)
IgA IF, > 10%	*Samples*	3/110 (2.7%)	2/82 (2.4%)	1/20 (5%)	1/38 (2.6%)	1/37 (2.7%)	0/6 (0%)
IgA Loc, all LPs	*mg/l*	0 (0-2.9; 106)	0 (0-2.9; 79)	0 (0-2; 19)	0 (0-2.9;36)	0 (0-0.5; 36)	0 (0-0; 6)
IgA Loc, QIgA positives	*mg/l*	0.5 (0.2-2.9; 5)	0.5 (0.2-2.9; 4)	2 (2-2; 1)	1.6 (0.4-2.9; 2)	0.4 (0.2-0.5; 2)	n.a. (n.a.; 0)
IgA CSF	*mg/l*	3.6 (0-40.7; 109)	3.57 (0-40.7; 81)	4.4 (0-29.1; 19)	4.34 (0-40.7; 36)	3.1 (0-7.99; 38)	3.4 (0-10.4; 6)
IgA serum	*g/l*	1.95 (0.25-7; 108)	1.8 (0.62-7; 81)	2.3 (1.1-3; 19)	1.77 (0.62-7; 37)	2.04 (0.73-5.16; 37)	1.94 (0.67-3.99; 6)

Quotients, concentrations, and fractions are given as median and range

*QIgG/A/M* CSF/serum IgG/A/M ratio; *IgG/A/M IF* intrathecally produced IgG/IgA/IgM fraction; *IgG/A/M loc* locally (intrathecally) produced IgG/A/M; *LP* lumbar puncture

Median IgM serum concentrations did not differ significantly between acute samples and samples obtained during remission (Table 3).

### Intrathecal IgA synthesis

QIgA was increased in just 5/111 (5%) samples (median QIgG 2.6; range 1.7-6.2) from 5 patients, with no difference between patients with acute ON and those with acute myelitis at the time of LP (5.3% vs. 5.3%) (Table 3 and Fig. 1). Among patients with elevated QIgA, the fraction of intrathecally produced IgA varied between 3.8 and 34.2% (median, 13.3), corresponding to an absolute amount of intrathecally produced IgA between 0.2 and 2.9 mg/l (median, 0.5). Median IgA CSF concentrations and IgA CSF/serum ratios were significantly higher (*p* < 0.00008 and *p* < 0.008, respectively) in the acute MY subgroup than in the acute ON subgroup (Table 3 and Fig. 1).

Median IgA concentrations in the serum did not differ significantly between acute samples and samples obtained during remission (Table 3 and Fig. 2).

### Immunoglobulin (Ig) class patterns

Only 1 out of 107 (0.9%) samples and 1 out of 75 (1.3%) patients tested exhibited a so-called three-class immune response as defined by elevation of QIgG, QIgM, and QIgA (no data in the remainder). While this particular sample was positive for a three-class reaction also according to a stricter definition based on Ig-IF > 10%, it was negative for OCB, strongly suggesting that the CSF/serum Ig ratios were falsely positive in this case (probably owing to plasma exchange [PEX] prior to LP; date of PEX commencement not precisely documented).

A two-class reaction defined by either positive QIgG and QIgM, positive QIgM and QIgA, or positive QIgG and QIgA was detected in only 5/107 (4.7%) samples and 4/75 (5.3%) patients tested based on QIg > Q_lim_(Ig) (Table 4). In 2 of these 5 patients (i.e., in just 2 out of 107 [1.9%] samples) a dominant IgG two-class reaction was noted; in 2 a dominant IgM reaction; and in 1 a dominant IgA response. If the stricter definition based on Ig-IF > 10% is used, the number of samples with a positive two-class reaction drops to 1/107 (0.9%) (IgM-dominant) (Table 4). By contrast, an IgG-dominant two-class response has been reported to occur in 20-40% of cases in MS [34, 35].

**Table 4:** Immunoglobulin class response patterns in MOG-IgG-positive EM

	Units	Total	Attack	Remission	Acute MY subgroup	Acute ON subgroup	Acute BRAIN subgroup
*a. Based on QIg > Q*_*lim*_*(Ig)*							
3-class reaction	Samples	1/107 (0.9%)	1/81 (1.2%)	0/19 (0%)	1/38 (2.6%)	0/36 (0%)	0/6 (0%)
2-class reaction	Samples	5/107 (4.7%)	4/81 (4.9%)	1/19 (5.3%)	2/38 (5.3%)	2/36 (5.6%)	0/6 (0%)
IgG + IgM	Samples	3/107 (2.8%)	3/81 (3.7%)	0/19 (0%)			
IgG + IgA	Samples	2/107 (1.9%)	1/81 (1.2%)	1/19 (5.3%)			
IgM + IgA	Samples	0/107 (0%)	0/81 (0%)	0/19 (0%)			
1-class reaction	Samples	15/107 (14%)	15/81 (18.5%)	0/19 (0%)	10/38 (26.3%)	3/36 (8.3%)	2/6 (33.3%)
Only IgG	Samples	4/107 (3.7%)	4/81 (4.9%)	0/19 (0%)			
Only IgM	Samples	9/107 (8.4%)	9/81 (11.1%)	0/19 (0%)			
Only IgA	Samples	2/107 (1.9%)	2/81 (2.5%)	0/19 (0%)			
*b. Based on Ig-IF > 10%*							
3-class reaction#	Samples	1/107 (0.9%)	1/81 (1.2%)	0/19 (0%)	1/38 (2.6%)	0/36 (0%)	0/6 (0%)
2-class reaction^§^	Samples	1/107 (0.9%)	1/81 (1.2%)	0/19 (0%)	1/38 (2.6%)	0/36 (0%)	0/6 (0%)
IgG + IgM	Samples	1/107 (0.9%)	1/81 (1.2%)	0/19 (0%)			
IgG + IgA	Samples	0/107 (0%)	0/81 (0%)	0/19 (0%)			
IgM + IgA	Samples	0/107 (0%)	0/81 (0%)	0/19 (0%)			
1-class reaction	Samples	9/107 (8.4%)	8/81 (9.9%)	1/19 (5.3%)	3/38 (7.9%)	4/36 (11.1%)	1/6 (16.7%)
Only IgG	Samples	3/107 (2.8%)	3/81 (3.7%)	0/19 (0%)			
Only IgM	Samples	4/107 (3.7%)	4/81 (4.9%)	0/19 (0%)			
Only IgA	Samples	2/107 (1.9%)	1/81 (1.2%)	1/19 (5.3%)			

^#^IF in the single sample with a three-class reaction: IgG-IF 18.79%, IgM-IF 21.65% and IgA-IF 31%

^§^IF in the single sample with a two-class reaction: IgG-IF 33.09% and IgM-IF 59%

Intrathecal Ig synthesis was restricted to one immunoglobulin class in 15/107 (14%) samples (IgG in 4; IgM in 9; IgA in 2) from 12 patients based on Ig CSF/serum ratios and in 9/107 (8.4%) samples based on Ig-IF > 10% (Table 4).

In two patients with intrathecal IgM and/or IgA synthesis but no quantitative evidence of intrathecal IgG synthesis, qualitative evidence for intrathecal IgG synthesis, i.e., CSF-restricted OCB, were detectable.

### MRZ reaction

**Fig. 5:**
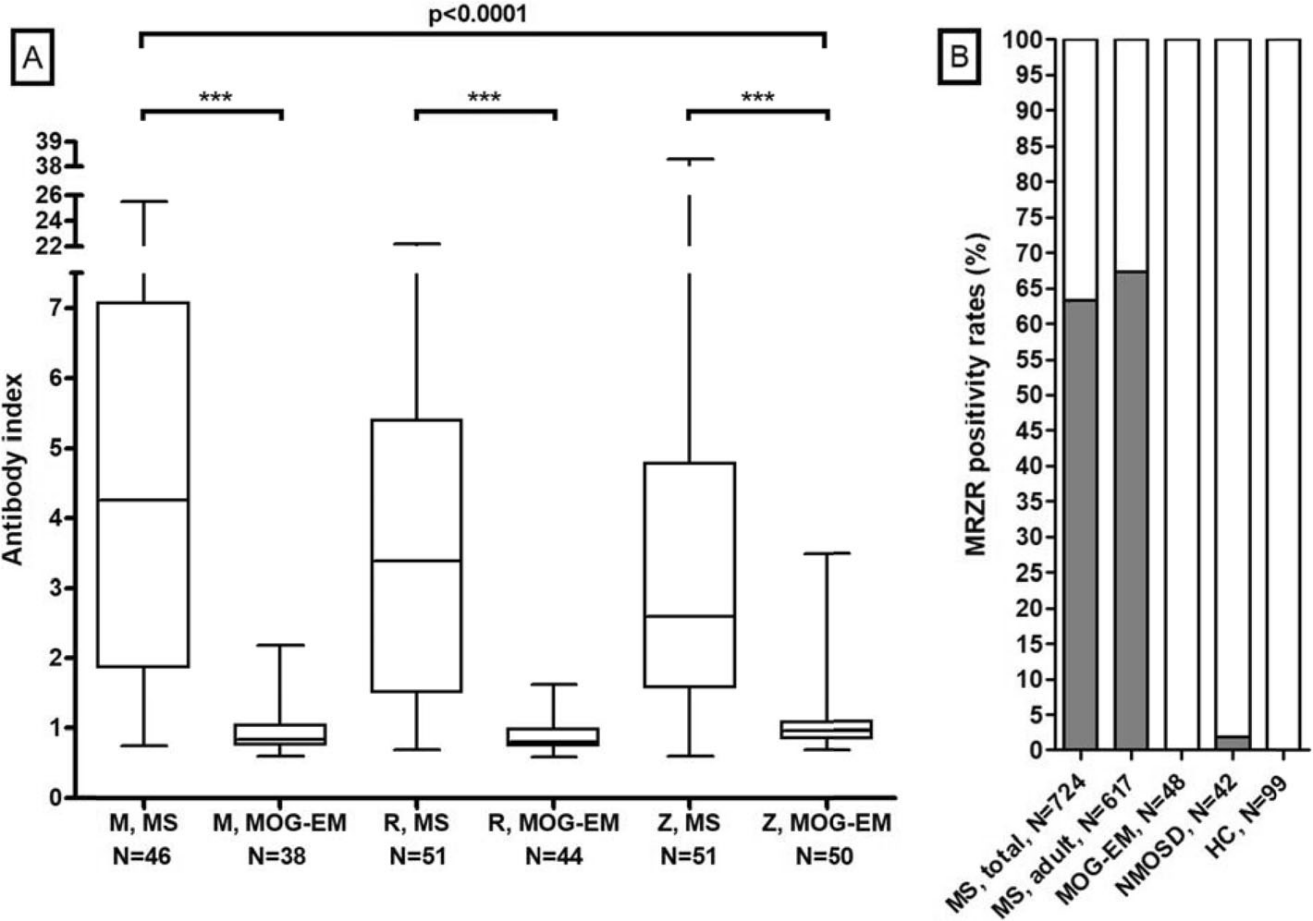
MRZ reaction. **a** The antibody indices for M, R, and Z in multiple sclerosis (pooled data from ref. [36, 38]) and in samples from MOG-IgG-positive patients (present study). Groups were compared using the Kruskal–Wallis test with Dunn’s post test. **b** The frequency of a positive MRZ reaction (MR, MZ, RZ, or MRZ) in MOG-EM (present study), in neuromyelitis optica spectrum disorders (NMOSD), and in healthy controls (HC) (data from [36]). AI, antibody index; M, measles virus AI; R, rubella virus AI; Z, varicella-zoster virus AI

Results of measles virus (M), rubella virus (R), and varicella-zoster virus (Z) antibody index (AI) determination were available for 61, 52, and 76 samples, respectively. All three AI were tested in 46 samples and two AI in 16 further samples. A positive MRZR, as defined by the presence of a positive IgG AI for at least two of its three constituents M, R and Z (i.e., by any of the following combinations: MR, MZ, RZ, or MRZ), is detectable in around 63% of cases in MS [36]. By contrast, the MRZ reaction was absent in 62/62 (100%) samples from 48 MOG-IgG-positive patients with available data (*p* < 0.000001 when compared to data from a reference paper on MRZR in MS [37]) (Table 5 and Fig. 5).

**Table 5:** Antibody indices

	Units	Total cohort
MRZ reaction (M + R, M + Z, R + Z, or M + R + Z)	*Patients*	0/48 (0%)
MRZ reaction (M + R, M + Z, R + Z, or M + R + Z)	*Samples*	0/62 (0%)
AI measle virus (M)	*Samples*	2/61 (3.3%)
AI rubella virus (R)	*Samples*	1/52 (1.9%)
AI varizella zoster virus (Z)	*Samples*	3/76 (3.9%)
Other antibody indices		
AI HSV	*Samples*	3/56 (5.4%)
AI EBV	*Samples*	1/18 (5.6%)
AI CMV	*Samples*	0/26 (0%)
AI *B. burgdorferi*, IgG	*Samples*	1/77 (1.3%)
AI *B. burgdorferi*, IgM	*Samples*	0/74 (0%)

MRZ reaction and antibody indices for measles virus (M), rubella virus (R), varicella zoster virus (V), herpes simplex virus (HSV), Epstein Barr virus (EBV), cytomegalovirus (CMV), and *Borrelia burgdorferi* (BB)

Intrathecal production of antibodies to measles (with or without concomitant antibodies against rubella and zoster virus) is the most common intrathecal antiviral immune response in MS, both in adults and in children [37]. While it is present in up to 86% of patients with MS, it was present in only 2/61 (3.3%) MOG-IgG samples (*p* < 0.000001 when compared to data from [37]; *N* = 177). A positive rubella virus AI was found for only 1/52 (1.9%) samples (*p* < 0.000001 vs. MS [37]), and a positive varicella-zoster virus AI for 3/76 (3.9%) (*p* < 0.000001 vs. MS [37]). However, no patient was positive for more than one of these AI.

Median AI for M, R, and Z were significantly lower in patients with MOG-EM than those in two previously published cohorts of patients with MS (*p* < 0.00001) (Fig. 5).

The MRZ reaction was negative not only in OCB-negative samples but also in 7/7 (100%) OCB-positive samples (from 6 different patients).

### Other antibody indices

A positive IgG AI (AI = 3.2) for *Borrelia burgdorferi* was present only in 1/77 (1.3%) samples from 65 patients tested. The *Borrelia* IgM AI was negative in 74 samples from 62 patients tested, including the Borrelia IgG AI-positive sample. None of the 26 samples tested exhibited a positive cytomegalovirus (CMV) IgG AI. A positive IgG AI for Epstein–Barr virus was noted in a single sample, while 17 other samples tested were negative. A positive IgG AI for herpes simplex virus (HSV) was found with 3/56 (5.4%) samples from 3 patients. In none of the Borrelia−, EBV−, or HSV-AI-positive patients, other AIs were reported to be elevated (although not all AIs were tested in all patients). See also Table 5.

### Blood–CSF barrier integrity

An elevated CSF/serum ratio for albumin, indicating dysfunction of the BCB, was found with 67/139 (48.2%) samples and was present at least once in 48/88 (54.5%) patients tested for this marker. QAlb ranged between 5.5-32.67 (median 9.6) (Table 6). Although absolute QAlb values decreased over time after an attack both in the total cohort (*r*^2^ = 0.055, *p* < 0.02) and in the acute myelitis subgroup (*r*^2^ = 0.126, *p* < 0.02) (Fig. 3), BCB dysfunction remained present during remission at almost the same frequency as during acute attacks (43.5% vs 46.7%; Table 6).

**Table 6:** Blood CSF barrier function, CSF total protein and CSF L-lactate in MOG-IgG-positive EM

	Units	Total	Attack	Remission	Acute MY subgroup	Acute ON subgroup	Acute BRAIN subgroup
Blood-CSF barrier function							
QAlb > QAlb(lim)	*Samples*	67/139 (48.2%)	49/105 (46.7%)	10/23 (43.5%)	28/50 (56%)	16/46 (34.8%)	4/8 (50%)
QAlb, all LPs	*-*	6.6 (3-32.7; 138)	6.4 (3-32.7; 105)	6 (3.1-18.5; 23)	7.4 (3.1-32.7; 50)	5.4 (3-26.3; 46)	7.8 (3.6-8.9; 8)
QAlb, if positive	*-*	9.6 (5.5-32.67; 66)	9.2 (5.5-32.67; 49)	11.8 (8.08-18.51; 10)	10.8 (6.4-32.7; 28)	7.8 (5.5-26.3; 16)	8 (6.8-8.9; 4)
Alb CSF	*mg/l*	273 (123-1260; 131)	261.5 (123-1260; 98)	280 (123-694; 22)	305 (133-1038.9; 46)	218 (123-1260; 43)	310 (175-371; 8)
Alb serum	*g/l*	42.1 (25.9-50.6; 126)	42.15 (25.9-50.6; 94)	41.8 (33.5-49.8; 22)	42.2 (25.9-50.6; 46)	42.2 (32.4-50.4; 39)	41.9 (37.3-48.7; 8)
Albumin-cellular dissociation	*Samples*	23/67 (34.3%)	14/49 (28.6%)	4/10 (40%)	2/28 (7.1%)	11/16 (68.8%)	1/4 (25%)
Combined BCB disruption							
and intrathecal IgG synthesis	*Samples*	12/67 (17.9%)	8/49 (16.3%)	1/10 (10%)	6/28 (21.4%)	1/16 (6.3%)	1/4 (25%)
CSF total protein							
CSF TP, elevated	*Samples*	64/146 (43.8%)	49/110 (44.5%)	9/25 (36%)	27/51 (52.9%)	16/50 (32%)	6/8 (75%)
CSF TP, all LPs	*mg/dl*	43.75 (5.11-176; 136)	43.75 (20.4-176; 102)	40 (5.11-148; 23)	49.6 (20.5-176; 48)	37.2 (20.4-171.8; 46)	48.2 (30.4-74.2; 8)
CSF TP, if elevated	*mg/dl*	64.65 (45.3-176; 64)	65 (45.3-176; 49)	61.9 (47-148; 9)	73.3 (46.7-176; 27)	60.4 (45.3-171.8; 16)	50.7 (47-74.2; 6)
CSF TP, > 100 mg/dl	*Samples*	11/136 (8.1%)	8/102 (7.8%)	2/23 (8.7%)	7/48 (14.6%)	1/46 (2.2%)	0/0 (0%)
CSF l-lactate							
CSF l-lactate, elevated	*Samples*	28/107 (26.2%)	25/79 (31.6%)	1/20 (5%)	18/37 (48.6%)	7/39 (17.9%)	0/3 (0%)
CSF l-lactate, all LPs	*mmol/l*	1.8 (1.05-4.43; 103)	1.9 (1.05-4.43; 76)	1.7 (1.33-2.4; 19)	2.31 (1.12-4.43; 35)	1.75 (1.05-2.68; 38)	1.51 (1.5-1.7; 3)
CSF L-lactate, if elevated	*mmol/l*	2.68 (2.1-4.43; 28)	2.68 (2.1-4.43; 25)	2.4 (2.4-2.4; 1)	2.53 (1.88-4.43; 20)	1.7 (1.05-2.68; 10)	1.51 (1.5-1.51; 2)
CSF l-lactate, >3 mmol/l	*Samples*	10/103 (9.7%)	8/76 (10.5%)	0/19 (0%)	8/35 (22.9%)	0/38 (0%)	0/3 (0%)

Ratios and concentrations are given as median (with ranges and sample numbers in brackets)

*Alb* albumin; *BCB* blood–CSF barrier; *LP* lumbar puncture; *QAlb* CSF/serum albumin ratio; *TP* total protein

In 23 samples (from 19 different patients) of 67 tested (34.3%), an albumin–cellular dissociation (ACD), i.e., compromised integrity of the BCB in the absence of CSF pleocytosis, was found. ACD was more commonly seen during remission (*p* = n.s.), reflecting the fact that pleocytosis was present more often during the acute phase and that BCB disruption was present also during remission in many cases (Table 6).

The frequency of BCB dysfunction was higher during acute MY attacks (56% [28/50]) than during acute ON attacks (34.8% [16/46]) (*p* < 0.05) (Table 6). In patients with acute myelitis, QAlb was positively linked to the spinal cord lesion load as detected by MRI and measured in VS (*r* = 0.386, *p* < 0.02) (Fig. 4).

### Total CSF protein

Total protein (TP) concentrations in the CSF were elevated in 64/146 (43.8%) samples (median 64.65 mg/dl; range 45.3-176) and at least once in 46/95 (48.4%) patients with available data. As expected, were significantly related to QAlb as detected by regression analysis (r2=0.874, *p*&lt;0.00001) (Supplementary Figure 3). Accordingly, QAlb was elevated in 85.2% of samples with increased CSF TP levels and available data on both parameters. Elevated CSF TP levels were > 45 and < 50 mg/dl (“borderline”) in 14/64 (21.9%) samples, ≥ 50 and ≤ 100 mg/dl in 39/64 (60.9%), between > 100 and ≤ 150 mg/dl in 9/64 (14.1%), and exceeded 150 mg in 2/64 (3.1%). Like QAlb, CSF TP levels were negatively correlated with the time (in days) since the onset of the last attack both in the total cohort (*r*^2^ = 0.061, *p* < 0.02) and in the acute myelitis subgroup (*r*^2^ = 0.117, *p* < 0.02) (Fig. 3). However, CSF TP levels were elevated not only during acute attacks (49/110 or 44.5%) but rather frequently also during remission (9/25 or 36%) (Table 6), which is in line with the fact that QAlb remained elevated during remission in many cases as well. Like QAlb, CSF TP levels were more commonly elevated in the acute MY subgroup than in the acute ON subgroup and was negatively correlated with time since attack onset (*r*^2^ = 0.117, *p* < 0.02) (Table 6); also, median CSF TP levels were higher in the MY subgroup than in the acute ON subgroup (*p* < 0.009) (Fig. 1).

### CSF l-lactate

Lactate levels were increased in 28/107 (26.2%) CSF samples (and at least once in 21/71 [29.6%] patients tested), with a median concentration of 2.68 mmol/l (range 2.1-4.43). Elevated lactate levels were found more frequently during acute attacks than during remission (31.6% vs. 5%; *p* = 0.020) (Table 6 and Fig. 2).

**Fig. 6:**
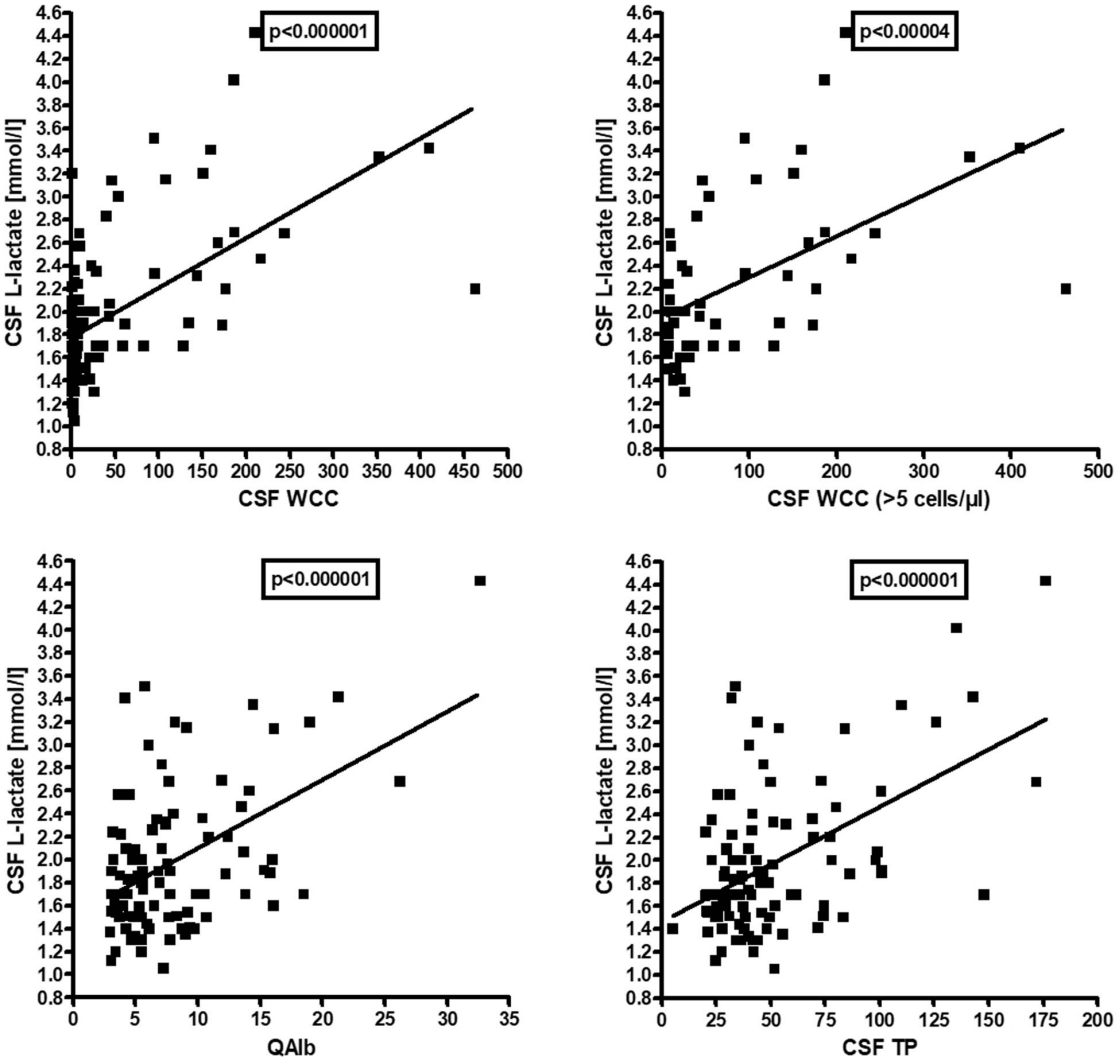
Correlation of CSF l-lactate concentrations with CSF WCC in the total cohort (upper left panel), with CSF WCC in patients with pleocytosis (upper right panel), QAlb (lower left panel) and QIgG (lower right panel). QAlb, albumin CSF/serum ratio, QIgG, IgG CSF/serum ratio; WCC, white cell count

Elevation of lactate levels was also significantly more common in the acute MY subgroup than in the acute ON subgroup (48.6% vs. 17.9%) (*p* < 0.007), and CSF lactate concentrations were significantly higher in the MY subgroup (*p* < 0.0003) (Fig. 1). Importantly, CSF lactate concentrations were significantly correlated with spinal cord lesion load in patients with acute myelitis (*r* = 0.646, i < 0.00009) (Fig. 4). We also found a significant positive correlation of l-lactate with the CSF WCC (both in the total cohort [*r* = 0.583, *p* < 0.0000001] and in patients with pleocytosis [*r* = 0.529, *p* < 0.00004]), with QAlb (*r* = 0.503, *p* < 0.0000001) and with CSF total protein (*r* = 0.513, *p* < 0.0000001) (Fig. 6). CSF l-lactate was elevated in only 7.8% (4/51) samples without pleocytosis but in 42.9% (24/56) of samples with pleocytosis, in 73.9% (17/23) of samples if CSF WCC exceeded 50 cells/μl, and in 82.4% (14/17) if CSF WCC exceeded 100 cells/μl. The difference was even more pronounced in the 'acute MY' subgroup (0% vs. 52.9%, 76.2%, and 86.7%, respectively). In patients with pleocytosis, the frequency of samples with elevated CSF l-lactate did not differ between samples with or without neutrophil granulocytes, neither in the total cohort (41.9% [18/43] vs. 42.9% [24/56]) nor in the 'acute MY' subgroup (52.2% [12/23] vs. 52.9% [18/34]). This renders it at least unlikely that granulocytes were the main source of l-lactate in patients with elevated CSF l-lactate levels. Accordingly, no significant correlation was found between CSF l-lactate levels and neutrophil cell numbers in the small subgroup (*N* = 19) of samples with available data (as well as in the 'acute MY' subgroup and the subgroup of samples with pleocytosis).

### First vs. follow-up LP

The frequency of OCB was slightly higher among the follow-up samples (11/58 [19%] vs. 9/93 [9.7%]). However, the difference was not statistically significant. All initial LP were obtained after 2002 (median 2015), largely ruling out the possibility that differences in IEF sensitivity between older and more recent tests played a major role. Moreover, the difference in OCB frequency was seen only in the acute MY subgroup, not in the acute ON or acute BRAIN subgroup. Finally, the frequency of IgG-IF elevation, as a quantitative marker for intrathecal IgG synthesis, did not differ between initial and follow-up samples. Given all these findings, it seems most likely that the difference in OCB frequency was by chance.

The frequency of pleocytosis, BCB dysfunction, CSF TP elevation, and CSF l-lactate elevation during acute attacks also did not differ significantly between the first LP and follow-up LP (Table 7).

**Table 7:** CSF findings at the time of the first LP and at the time of follow-up

	Units	First LP ever	Follow-up LPs, first LP/event
Pleocytosis, all acute attacks	*Samples*	44/79 (55.7%)	10/23 (43.5%)
Pleocytosis, acute MY	*Samples*	26/31 (83.9%)	6/9 (66.7%)
Pleocytosis, acute ON	*Samples*	12/39 (30.8%)	3/11 (27.3%)
Pleocytosis, acute BRAIN	*Samples*	5/7 (71.4%)	1/3 (33.3%)
OCB, all acute attacks	*Samples*	7/73 (9.6%)	2/23 (8.7%)
OCB, acute MY	*Samples*	1/27 (3.7%)	2/10 (20%)^#^
OCB, acute ON	*Samples*	3/38 (7.9%)	0/10 (0%)
OCB, acute BRAIN	*Samples*	2/7 (28.6%)	0/3 (0%)
IgG-IF > 10%, all acute attacks	*Samples*	4/63 (6.3%)	3/24 (12.5%)
IgG-IF > 10%, acute MY	*Samples*	2/26 (7.7%)	0/9 (0%)
IgG-IF > 10%, acute ON	*Samples*	2/31 (6.5%)	0/9 (0%)
IgG-IF > 10%, acute BRAIN	*Samples*	0/5 (0%)	0/3 (0%)
QAlb > Q_lim_(Alb), all acute attacks	*Samples*	35/65 (53.8%)	7/23 (30.4%)
QAlb > Q_lim_(Alb), acute MY	*Samples*	16/26 (61.5%)	6/10 (60%)
QAlb > Q_lim_(Alb), acute ON	*Samples*	14/33 (42.4%)	1/10 (10%)
QAlb > Q_lim_(Alb), acute BRAIN	*Samples*	4/5 (80%)	0/3 (0%)
CSF TP elevated, all acute attacks	*Samples*	33/70 (47.1%)	9/23 (39.1%)
CSF TP elevated, acute MY	*Samples*	15/27 (55.6%)	6/10 (60%)
CSF TP elevated, acute ON	*Samples*	14/37 (37.8%)	1/10 (10%)
CSF TP elevated, acute BRAIN	*Samples*	4/5 (80%)	2/3 (66.7%)
CSF l-lactate elevated, all acute attacks	*Samples*	14/52 (26.9%)	2/13 (15.4%)
CSF l-lactate elevated, acute MY	*Samples*	9/21 (42.9%)	1/5 (20%)
CSF l-lactate elevated, acute ON	*Samples*	5/28 (17.9%)	1/8 (12.5%)
CSF l-lactate elevated, acute BRAIN	*Samples*	0/3 (0%)	0/0 (0%)
Time since attack onset, acute LPs	*Days*	7 (0-34)	9.5 (0-28)

To control for the fact that the number of CSF samples obtained per event differed among patients in the subgroup with follow-up LPs, only the first LP obtained during each attack was taken into account for this analysis

*IgG-IF* intrathecal IgG fraction; *OCB* oligoclonal bands; *QAlb* CSF/serum albumin quotient; *TP* total protein; *WCC* white cell count

^#^p = n.s.

However, changes were noted in individual patients over time. A total of 51 repeat tests for OCB were performed in 33 patients. OCB turned negative in at least one repeat sample in 1 patient over the course of the disease (absent 1 week after the initial LP and following steroid treatment) (Supplementary Table 2). In 2 patients, OCB were negative at first LP and turned positive at repeat examination 271 and 66 days later, respectively. QIgG was normal in most of the OCB-positive samples, indicating low levels of IgG IS.

In 26 patients, OCB were initially negative and remained negative at follow-up. In 5 of these patients, OCB pattern changed from pattern 1 to pattern 4, or vice versa, over time. In three patients, OCB were positive at all LP performed (2 × 3 LP, 1 × 2 LP).

Similarly, QIgG was positive only transiently in 3/4 patients with quantitative evidence for IgG IS and available follow-up results. 24/28 (85.7%) patients who were tested more than once had a normal IgG CSF ratio both at first LP and at follow-up. An 'IgM to IgG IS switch' was only observed in 1/21 patients in whom QIgG and QIgM were both determined more than once (QIgG negative but QIgM positive at first LP, QIgG and QIgM positive at repeat LP a few days later and following treatment with dexamethasone and intravenous methylprednisolone).

In MRZR-negative patients, repeat lumbar puncture was reported to increase the sensitivity of MRZR for MS due to “broadening” of the MRZ reaction over time [39]. It is therefore of note that some of the sample used for MRZ testing were obtained at first LP (*N* = 40), whereas others were obtained at follow-up LP (*N* = 22); however, all were negative, irrespective of disease duration at the time of MRZ testing (median 117.5 days since onset of 1st attack; range 06885). Moreover, in 9 patients, MRZR was tested more than once. All 14 retests (1-5 per patient; median 1.5) in these patients were negative as well; the median latency between first and last MRZR testing was 766.5 days (range 10-2157).

### Attack severity

Most CSF parameters assessed were higher and/or more frequently pathologically altered in patients classified as having a severe attack at the time of LP by the treating physician than in patients classified as having mild or moderate disease at the time of LP (Table 8), including, among others, median CSF WCC (43.3 vs. 7 cells/μl; *p* < 0.0005); proportion of samples with pleocytosis (80% vs. 53%; *p* < 0.007); QIgG values (*p* < 0.012); QIgG positivity rate (13% vs. 7%; *p* = n.s.); OCB positivity rate (16% vs. 9%; *p* = n.s.); rate of Link index (i.e., IgG index) elevation (17% vs. 6%; *p* = n.s.); QAlb positivity rate (67% vs. 39%; *p* = 0.017); median QAlb (7.9 vs. 5.7; *p* = 0.009); median QAlb, if elevated (10.8 vs. 8.3; *p* < 0.014); median CSF TP levels (*p* = 0.026); and presence of CSF TP exceeding 100 mg/dl (20% vs. 4%; *p* < 0.009). CSF TP concentrations > 100 mg/dl or CSF l-lactate concentrations > 3 mmol/l were seen in none of the samples obtained from patients with a mild attack at the time of LP but were present in 20% and 24%, respectively, of patients with a severe attack. See Table 8 for details.

**Table 8:** CSF findings and attack severity

	Units	Severe attacks	Mild/moderate attacks	*p* value
WCC, all	*Cells/μl*	43.3 (0-410; 35)	7 (0-463;83)	0.0005
WCC, elevated	*Samples*	28/35 (80%)	44/83 (53%)	0.007
WCC, if elevated	*Cells/μl*	47 (6-410; 28)	24 (6-463;44)	n.s.
Neutrophils, all LPs	*Samples*	8/17 (47.1%)	18/40 (45%)	n.s.
OCB, pattern 2 or 3	*Samples*	5/32 (15.6%)	7/78 (9%)	n.s.
Link index	*Samples*	5/30 (16.7%)	4/68 (5.9%)	n.s.
QIgG, all	*Ratio*	4.2 (0.9-23.6; 30)	3.2 (1.1-14.1; 69)	0.012
QIgG, elevated	*Samples*	4/30 (13.3%)	5/69 (7.2%)	n.s.
QIgG, if elevated	*Ratio*	4.7 (2.3-6; 4)	4.5 (2.4-7; 5)	n.s.
QAlb, all	*Ratio*	7.9 (3.2-32.7; 30)	5.7 (3-26.3; 74)	0.009
QAlb, elevated	*Samples*	20/30 (66.7%)	29/74 (39.2%)	0.017
QAlb, if elevated	*Ratio*	10.8 (6.5-32.7; 20)	8.3 (5.5-26.3; 29)	0.014
CSF TP, all	*mg/dl*	55.1 (20.5-176; 31)	41.4 (20.4-171.8; 77)	0.026
CSF TP, elevated	*Samples*	18/31 (58.1%)	31/77 (40.3%)	n.s.
CSF TP, if elevated	*mg/dl*	79 (46.7-176; 18)	62 (45.3-171.8; 31)	0.021
CSF TP, > 100 mg/dl	*Samples*	6/30 (20%)	3/85 (3.5%)	0.009
CSF l-lactate, all	*mmol/l*	2 (1.4-4.4; 22)	1.8 (1.1_4; 56)	n.s.
CSF l-lactate, elevated	*Samples*	9/22 (40.9%)	16/56 (28.6%)	n.s.
CSF l-lactate, if elevated	*mmol/l*	3.1 (2.3-4.4; 9)	2.6 (2.1-4; 16)	0.037
CSF l-lactate, > 3 mmol/l	*Samples*	6/25 (24%)	4/60 (6.7%)	n.s.

Ratios and concentrations are given as median (with ranges and sample numbers in brackets)

*IgG-IF* intrathecal IgG fraction; *OCB* oligoclonal bands; *QAlb* CSF/serum albumin quotient; *TP* total protein; *WCC* white cell count

### LETM vs. non-longitudinally extensive transverse myelitis (NETM)

While a significant correlation of CSF l-lactate, QAlb, QIgG, and WCC with the total lesion load was found, as described above, no statistically significant differences regarding the frequency of CSF pleocytosis, CSF-restricted OCB, IF-IgG elevation > 10%, BCB dysfunction, or CSF TP elevation were noted when samples were stratified into acute LETM and acute NETM based on the presence or absence of at least one lesion extending over three or more VS (Supplementary Table 3). CSF l-lactate levels were slightly yet statistically significantly higher in LETM samples (*p* < 0.05).

### Bilateral vs. unilateral ON

CSF findings in acute bilateral ON and unilateral ON did not differ significantly, although more samples from patients with acute bilateral ON than samples from patients with acute unilateral ON exhibited an increased CSF WCC, a positive QAlb, or CSF TP elevation (Supplementary Table 4).

### Disease course

No statistically significant differences between samples from patients with a monophasic disease course at last follow-up and patients with a relapsing disease course were observed during acute attacks with regard to the frequency of CSF-restricted OCB, CSF pleocytosis, IgG-IF > 10%, QAlb elevation, CSF l-lactate elevation, and CSF TP elevation (Table 9).

**Table 9:** CSF findings in samples from patients with monophasic disease and from patients with relapsing disease

1st LP/event	Units	Monophasic	Relapsing
Pleocytosis, acute attacks	*Samples*	17/27 (63%)	37/74 (50%)^§^
Pleocytosis, acute MY	*Samples*	12/14 (85.7%)	20/26 (76.9%)
Pleocytosis, acute ON	*Samples*	3/9 (33.3%)	12/40 (30%)
Pleocytosis, acute BRAIN	*Samples*	2/4 (50%)	4/6 (66.7%)
OCB, acute attacks	*Samples*	4/25 (16%)	5/70 (7.1%)^§^
OCB, acute MY	*Samples*	1/12 (8.3%)	2/25 (8%)
OCB, acute ON	*Samples*	1/9 (11.1%)	2/38 (5.3%)
OCB, acute BRAIN	*Samples*	2/4 (50%)	0/6 (0%)
IgG-IF > 10%, acute attacks	*Samples*	1/21 (4.8%)	3/62 (4.8%)^§^
IgG-IF > 10%, acute MY	*Samples*	1/12 (8.3%)	1/23 (4.3%)
IgG-IF > 10%, acute ON	*Samples*	0/6 (0%)	2/33 (6.1%)
IgG-IF > 10%, acute BRAIN	*Samples*	0/3 (0%)	0/5 (0%)
QAlb > Q_lim_(Alb), acute attacks	*Samples*	12/21 (57.1%)	30/66 (45.5%)^§^
QAlb > Q_lim_(Alb), acute MY	*Samples*	8/12 (66.7%)	14/24 (58.3%)
QAlb > Q_lim_(Alb), acute ON	*Samples*	2/6 (33.3%)	13/36 (36.1%)
QAlb > Q_lim_(Alb), acute BRAIN	*Samples*	2/3 (66.7%)	2/5 (40%)
CSF TP elevated, acute attacks	*Samples*	15/24 (62.5%)	27/68 (39.7%)^§^
CSF TP elevated, acute MY	*Samples*	9/12 (75%)	12/25 (48%)
CSF TP elevated, acute ON	*Samples*	4/9 (44.4%)	11/37 (29.7%)
CSF TP elevated, acute BRAIN	*Samples*	2/3 (66.7%)	4/5 (80%)
CSF l-lactate elevated, acute attacks	*Samples*	7/18 (38.9%)	8/46 (17.4%)^§^
CSF l-lactate elevated, acute MY	*Samples*	6/10 (60%)	4/16 (25%)
CSF l-lactate elevated, acute ON	*Samples*	1/6 (16.7%)	4/29 (13.8%)
CSF l-lactate elevated, acute BRAIN	*Samples*	0/2 (0%)	0/1 (0%)
Time since attack onset, acute LPs	*Days*	5 (0-31)	9.5 (0-34)

To control for differences in the number of follow-up samples available per patient, only the first LP performed during each acute event was considered

*IgG-IF* intrathecal IgG fraction; *OCB* oligoclonal bands; *QAlb* CSF/serum albumin quotient; *TP* total protein

^§^*p* = n.s.

### Treatment status

No statistically significant differences were found between samples from patients untreated at the time of LP (*N* = 102) and samples from patients treated with steroids, immunosuppressants, or immunomodulatory drugs at the time of LP (*N* = 56). Steroids used included methylprednisolone, prednisone, and dexamethasone (*N* = 47); immunosuppressive or immunomodulatory treatments used included azathioprine, rituximab, fingolimod, intravenous immunoglobulins, dimethyl fumarate, teriflunomide, cyclophosphamide and were either used alone (*N* = 9) or in combination with steroids (*N* = 9). Surprisingly, pleocytosis, CSF-restricted OCB, elevated QIgG, BCB damage (as suggested by an elevated QAlb), and an increase in CSF TP concentrations were all found with equal or even higher frequency with samples from treated patients. The most likely explanation for these findings is that more patients with a severe disease course (and thus more pronounced CSF alterations, as shown above) received treatment than did patients with mild attacks (and thus less pronounced CSF alterations). In fact, 52% of all samples obtained during severe attacks, but only 33% of samples obtained during mild attacks, were samples obtained from treated patients. Moreover, patients with acute spinal cord or brain disease—and thus greater lesion volume and, in consequence, often more pronounced CSF alterations, as shown above—were more likely to receive treatment than patients with acute ON (ON samples accounted for only 34.8% of the 'treated acute samples' but for 43.1% of all 'acute samples'). Finally, patients with mild or moderate ON, the subgroup with the least probability of pathological CSF alterations, were underrepresented in the treated subgroup when compared with the total cohort, further strengthening the effect.

### OCB-positive vs. OCB-negative MOG-EM

OCB-positive patients did not differ significantly from OCB-negative patients with regard to age, sex, origin, or disease duration. Similarly, no marked differences were found between OCB-positive and OCB-negative samples regarding most CSF parameters, with the exception of a higher rate of pleocytosis (*p* = n.s.) and a slightly higher median WCC in the OCB-positive subgroup (*p* = n.s.), possibly indicating slightly more severe disease among those patients (Table 10).

**Table 10:** Demographic and CSF findings in MOG-EM stratified according to OCB status

		OCB+ patients (at least once positive)	OCB– patients (never positive)	
Sex (m:f)	Patients	1:1.5	1:1.5	
Origin (non-Caucasion:Caucasian)	Patients	1:14	1:11	
Median age at first OCB+ LP	Years	34	38	
Median disease duration at first OCB+ LP	Months	0 (0-16)	0 (0-489)	
		OCB+ all samples	OCB− all samples	
Pleocytosis	Samples	13/19 (68.4%)	57/128 (44.5%)	*p = n.s.*
WCC	Samples	14 (0-151)	4 (0-463)	*p = n.s.*
WCC > 150	Samples	1/18 (5.6%)	9/128 (7%)	*p = n.s.*
Granulocytes	Samples	3/10 (30%)	26/62 (41.9%)	*p = n.s.*
QAlb > Q_lim_(Alb)	Samples	11/17 (64.7%)	51/117 (43.6%)	*p = n.s.*
CSF TP elevated	Samples	8/18 (44.4%)	50/122 (41%)	*p = n.s.*
CSF l-lactate elevated	Samples	3/12 (25%)	22/91 (24.2%)	*p = n.s.*
Treated at the time of OCB testing	Samples	10/20 (50%)	48/131 (36.6%)	*p = n.s.*
Time since attack onset	Days	14	12.5	

*IgG-IF* intrathecal IgG fraction; *OCB* oligoclonal bands; *QAlb* CSF/serum albumin quotient; *TP* total protein; *WCC* white cell count

### Acute attacks vs. remission

A significantly higher frequency of pleocytosis (94.7% vs. 13.5%; *p* < 0.005), a significantly higher CSF WCC (*p* < 0.0007) and a significantly higher frequency of CSF l-lactate elevation (31.6% vs. 5%; *p* = 0.020) were found in CSF samples obtained during acute attacks compared to samples obtained during remission if all samples were taken into account (see Tables 1, 2, 3, 4, 6 and Fig. 2).

When considering only the first LP performed during acute events and the latest LP performed during remission of an event (in order to control for differences in the number of follow-up samples available per patient), some differences were more pronounced (Table 11 and Fig. 2). However, highly statistically significant differences were found only for pleocytosis rate (*p* < 0.0004) and CSF WCC numbers (*p* < 0.0002). A less marked difference was observed for QIgM (*p* < 0.03).

**Table 11:** CSF findings during acute attacks (first LP/event) and during remission (last LP/event)

	Units	Attack, all, first LP/event	Remission, all, last LP/event	*p* values
Pleocytosis	*Samples*	54/102 (52.9%)	2/20 (10%)	*p* = 0.0004
WCC	*Cells/μl*	6.5 (0-463; 100)	2 (0-16; 19)	*p* < 0.0002
WCC > 100/μl	*Samples*	13/101 (12.9%)	0/20 (0%)	*p* = n.s.
OCB	*Samples*	9/96 (9.4%)	2/20 (10%)	*p* = n.s.
QIgG > Q_lim_(IgG)	*Samples*	6/85 (7.1%)	1/18 (5.6%)	*p* = n.s.
QIgG	*Ratio*	3.47 (0.9-23.61; 84)	2.66 (1.47-6.7; 18)	*p* = n.s.
IgG-IF > 10%	*Samples*	4/84 (4.8%)	0/18 (0%)	*p* = n.s.
QIgM > Q_lim_(IgM)	*Samples*	11/73 (15.1%)	0/15 (0%)	*p* = n.s.
QIgM	*Ratio*	0.59 (0-13.81; 72)	0.3 (0-1.36; 15)	*p* < 0.03
QIgA > Q_lim_(IgA)	*Samples*	3/72 (4.2%)	1/15 (6.7%)	*p* = n.s.
QIgA	*Ratio*	1.76 (0-17.27; 71)	1.94 (0-4.83; 15)	*p* = n.s.
QAlb > Q_lim_(Alb)	*Samples*	42/88 (47.7%)	5/18 (27.8%)	*p* = n.s.
QAlb	*Ratio*	6.46 (2.98-32.67; 88)	5.55 (3.13-15.33; 18)	*p* = n.s.
CSF TP elevated	*Samples*	42/93 (45.2%)	5/20 (25%)	*p* = n.s.
CSF TP concentration	*mg/dl*	44.5 (20.5-176; 85)	36.65 (5.11-101; 18)	*p* = n.s.
CSF TP > 100 mg/dl	*Samples*	7/93 (7.5%)	1/20 (5%)	*p* = n.s.
CSF l-lactate elevated	*Samples*	16/65 (24.6%)	0/15 (0%)	*p* = 0.033
CSF l-lactate concentration	*mg/dl*	1.82 (1.05-4.43; 62)	1.7 (1.33-2.1; 14)	*p* = n.s.
CSF l-lactate > 3 mmol/l	*Samples*	4/65 (6.2%)	0/15 (0%)	*p* = n.s.
Time since attack onset	*Days*	8 (0-34; 104)	191 (117-2624; 20)	

Ratios and concentrations are given as median (with ranges and sample numbers in brackets)

*IgG-IF* intrathecal IgG fraction; *OCB* oligoclonal bands; *QAlb* CSF/serum albumin quotient; *TP* total protein

Finally, separate analyses of the 'acute MY', the 'acute ON', and the 'acute BRAIN' subgroups revealed more pronounced differences between the acute phase and remission than is evident from the unstratified analysis of the total cohort (see Supplementary Table 1, Table 11, and Fig. 2).

### 'Normal' CSF

A substantial number of CSF samples exhibited no pathological changes. If CSF WCC, OCB, QIgG, Link index, QIgM, QIgA, QAlb, CSF TP, and CSF l-lactate are taken into account 15/163 (9.2%) samples showed exclusively normal values. Of these 15 samples, 5 were taken during remission and 10 during acute attacks. Most of the 'acute' samples were obtained from patients with ON (*N* = 7). If only a basic panel consisting of CSF WCC, CSF TP, and CSF l-lactate is considered (reflecting clinical practice in some non-tertiary centers and in emergency room settings), 34/163 (21%) samples would have been classified as “normal.” Of those, 19 (56%) would have been false-negatives (with the full panel serving as a gold standard).

### Quotient diagrams (“reibergrams”)

**Fig. 7:**
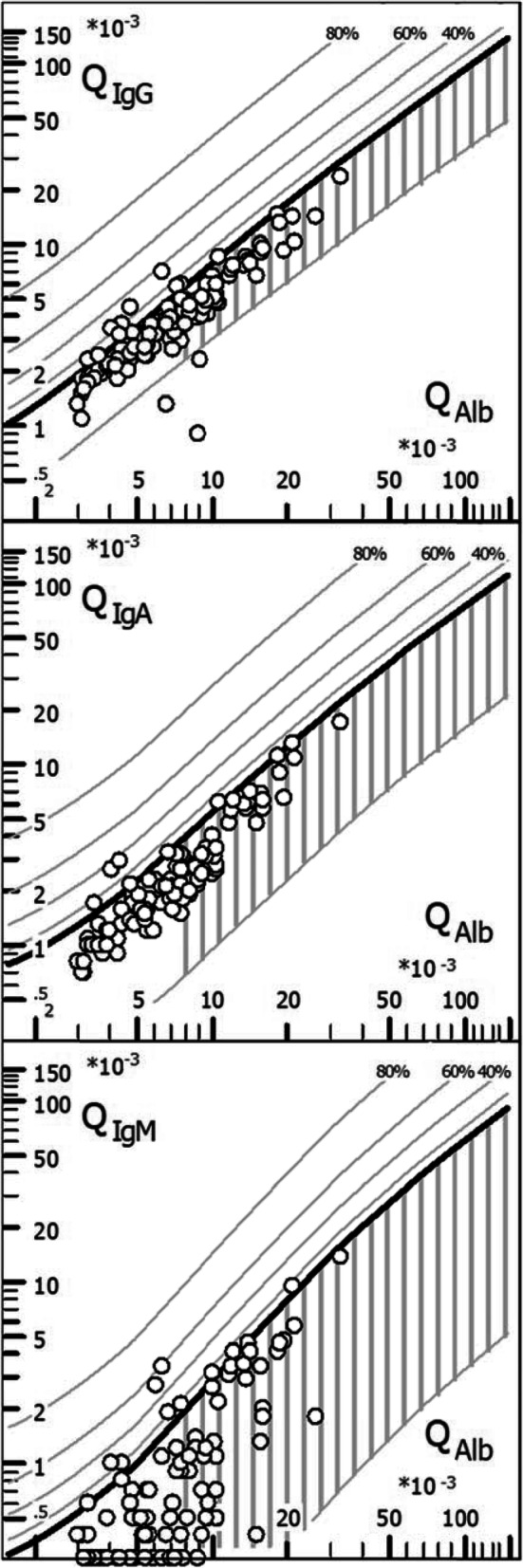
CSF/serum quotient diagrams for IgG, IgM, and IgA (“reibergrams”). Individual CSF/serum ratios of IgG, IgA, and IgM are plotted against CSF/serum albumin ratios. Values above the upper hyperbolic discrimination line, Q_lim_, indicate intrathecal synthesis of the respective immunoglobulin (Ig) class. Individual intrathecal fractions, Ig_IF_, can be directly read by interpolation from the percentiles above Q_lim_ (median values are given in Tables 2 and 3). IgG/A/M, immunoglobulin G/A/M; QIgG/A/M, CSF/serum IgG/A/M ratios; QAlb, CSF/serum albumin ratio

Plots of QIgG, QIgA, and QIgM, respectively, against QAlb as a measure of BCB function are shown in Fig. 7.

## Discussion

In this study, which is the largest and most comprehensive study on CSF findings in MOG-EM conducted to date, we demonstrate that CSF findings in MOG-EM are clearly different from those reported in MS [27, 40]. Our findings add further evidence in favor of the hypothesis that MOG-IgG-positive EM is a disease in its own right rather than a subvariant of MS [41–44].

Most strikingly, 131/151 (87%) samples showed no signs of intrathecal synthesis (IS) of IgG, as indicated by a lack of CSF-restricted OCB. This is in stark contrast to MS, in which OCB are detectable in ≥ 95% of cases [27, 40]. In those samples positive for OCB, the amount of intrathecally produced IgG was mostly low, as indicated by normal QIgG in 10/17 samples. In the few samples with quantitative evidence of intrathecal IgG synthesis (i.e., with elevated QIgG), the intrathecal IgG fraction was below the second decile in the IgG-specific reibergram in 7/10 (70%) cases and was even below 10% in 5/10 (50%). Moreover, quantitative evidence of intrathecal total IgG synthesis, if present at all, was found exclusively during acute disease attacks and was present only transiently in 3/4 patients with available follow-up data. Similarly, OCB were only transiently positive in 3/6 OCB-positive patients tested more than once (Supplementary Table 2). This is in contrast to the temporal invariance of intrathecal IgG synthesis deemed typical for MS [45], again suggesting a differential immunopathogenesis of the two disorders. Temporal variance of the patient’s OCB status has also been observed in patients with AQP4-IgG-positive NMOSD [15, 46].

The specificity of the intrathecally produced IgG fraction in the few OCB-positive patients with MOG-EM is unknown. MOG-IgG have been previously reported to be present in the CSF in a subset of patients with MOG-EM [47–49]. However, MOG-IgG is primarily produced in the periphery, i.e., extrathecally, as suggested by a negative MOG-specific AI [2]. This is similar to AQP4-IgG-positive NMOSD, in which the pathogenic antibody is also predominantly produced extrathecally [46, 48–50]. While the latter fact does not rule out the possibility that a small proportion of MOG-IgG is produced intrathecally, it is unlikely that intrathecally produced MOG-IgG contributes much to the intrathecally produced IgG fraction detectable in some patients, since antigen-specific AI are generally considered to be more sensitive than OCB and QIgG (which measure total IgG). Alternatively, the intrathecally produced IgG could reflect secondary B cell activation, e.g., targeted at antigens unmasked by primary inflammatory tissue damage. Finally, it might be related to coexisting conditions in some patients. Connective tissue disorders (CTD), for example, which relatively frequently co-exist with AQP4-IgG-positive NMOSD [51–53], are associated with OCB in neurological patients in about 25-30% of cases [54, 55]. However, signs of CTD were documented in none of the OCB-positive MOG-EM patients in the present study (except for ANA). OCB can be observed also in CNS infection. Similar to NMOSD and MS, attacks in MOG-EM have been indeed reported to be preceded by viral or bacterial infections (or vaccination) in up to 30% of cases [56]. It is therefore of note that OCB patterns 3 and 4, indicating possible systemic infection at the time of LP, were present in 33/150 (22%) samples. Moreover, three OCB-positive patients showed a single positive anti-viral AI (2 × VZV, 1 × EBV), one OCB-positive patient suffered from possible zoster thoracicus 2-3 weeks before onset of disease and LP (interestingly, an episode of possible herpes zoster preceded disease onset and LP also in a second, though OCB-negative, patient in this cohort by 2-3 weeks), and one had a history of borreliosis.

Importantly, the intrathecal, polyspecific antiviral IgG response typically found in MS (also termed MRZ reaction) was absent in all samples tested (*N* = 62). This is similar to what has been reported in NMOSD [37, 38, 57]. By contrast, a positive MRZ reaction is detectable in around 60-70% of patients with classic MS [36, 37]. The lack of MRZ in patients with MOG-EM, one of the most important differential diagnoses of MS, adds to previous evidence indicating a very high specificity of the MRZ reaction for MS. The MRZ reaction is currently considered the laboratory marker with the highest positive likelihood ratio for MS [36, 38, 57, 58]. Its absence in MOG-IgG-positive patients strongly supports the notion that MS and MOG-EM are two pathophysiologically distinct diseases (Fig. 5).

Finally, the possibility of false-positive results needs to be taken into account. QIgG results should be interpreted with caution whenever IgG-IF values are below 10%, owing to the limited precision of IgG measurements in serum and CSF, which are inherent to the methods (nephelometry) used, if supporting evidence from OCB determination (which, performed properly, is substantially more sensitive than QIgG) is lacking. Current guidelines on CSF diagnosis set the upper limit for imprecision at 7-10%, for incorrectness at 10%, and for deviation between single measures at 24-30% [30]. In fact, IgG-IF was below 10% and OCB were negative or were not tested in 3 samples from 3 patients. If QIgG results not supported by either an IF-IgG > 10% or CSF-restricted OCBs are not considered true positive (as recommended by some authors [30]), QIgG elevation was present only in 8/133 (6%) samples from 6 out of 88 (6.8%) patients tested for QIgG at least once (including the two VZV-AI-positive patients), indicating that IS of IgG to an extent that is detectable quantitatively is rare in MOG-EM. If the two VZV-AI-positive patients are excluded as well (since intrathecal VZV infection may sufficiently explain QIgG elevation), only 4 patients showed an elevated QIgG, in at least two of whom QIgG was positive only transiently.

There is still no standardized assay for detecting MOG-IgG. Given the rarity of MOG-EM, very high assay specificity is required to avoid an unfavorable ratio of false-positive to true-positive results. However, none of the assays published so far has been evaluated in sufficiently large control cohorts to meet that requirement. As in our previous studies on MOG-IgG [2–5]—and different from most studies in the field—the present study therefore included almost exclusively patients tested positive for MOG-IgG in at least two methodologically independent assays, resulting in high diagnostic accuracy and diagnostic homogeneity of this cohort and thus improved data quality/validity. We consider this one of the strengths of the present analysis. For confirmation, samples were retested by means of up to four different assays performed in two different laboratories (Medical University Innsbruck: H + L-specific live CBA, Fc-specific live CBA; University of Heidelberg: Fc-specific fixed CBA, IgG1-specific live CBA). Overall, 13 patients from 5 centers proposed for inclusion were not included in the study because re-testing did not confirm MOG-IgG seropositivity. These samples were thus classified as false positives. In all of these cases, a borderline or only low-positive test result had been reported by the initially testing laboratory. It is interesting in the present context that 4 of the false positives had shown a positive MRZ reaction, as typically seen in MS, one of whom had a primary-progressive disease course, which is atypical in MOG-EM but not infrequent in MS, and three of whom had a relapsing-remitting disease course; all four met the current diagnostic criteria for MS. Together with the complete absence of a positive MRZ reaction among patients with confirmed MOG-IgG serostatus in our study, this strongly suggests—in accordance with recent international recommendations on the diagnosis of MOG-EM [22, 59]—that a positive MRZ reaction should be considered a diagnostic 'red flag', i.e., a finding that should prompt physicians to critically challenge a “positive” MOG-IgG laboratory report.

Few (*N* = 13, or 12% based on Q_lim_, and *N*=6, or 5%, based on IgM-IF > 10%) patients showed low intrathecal production of IgM antibodies. IgM IS was found exclusively during acute attacks. Interestingly, in 9/107 (8.4%) cases, IS exclusively of IgM but not of IgG was observed, whereas in the remaining four cases IgG and IgM IS was present in parallel. By contrast, isolated IgM IS is atypical in MS and should prompt doubt regarding that diagnosis. The specificity of the IgM antibodies in our MOG-EM patients is unknown. Five of the 13 CSF samples with elevated QIgM were available for retrospective testing but were all negative for MOG-IgG (testing performed after preabsorption of total IgG to rule out false-positive or false-negative results [60]). To the best of our knowledge, there are also no reports on marked IgM deposition in MOG-EM lesions (as seen in NMOSD) [61]. A previous study found MOG-IgM in 2/23 MOG-IgG-positive serum samples but did not test for MOG-IgM in the CSF [3]. Alternatively, blood contamination could have played a role in a subset of cases. QIgM is much more sensitive to blood contamination than QIgG, and a relevant number of erythrocytes were detectable in at least 2/13 QIgM-positive patients. Finally, the intrathecal IgM fraction was < 10% in 7/13 QIgM-positive patients. In patients with such low IF values, false-positive QIgM results (owing to unavoidable imprecision of IgM measurements [30]) cannot be fully ruled out.

Blood CSF barrier disturbance as indicated by QAlb elevation was common and more severe than in MS. While QAlb is normal in around 90% of MS patients [27, 33], it was elevated in almost every second MOG-EM sample. This is very similar to the high frequency of BCB disruption seen in AQP4-IgG-positive NMOSD (51%) [46]. Similarly, QAlb values exceeding 12 × 10^−3^, which are extremely rare in MS, were present in more than a quarter of samples with elevated QAlb. This is of importance, since extrathecally produced MOG-IgG might gain access to the CNS via regions of disturbed BCB permeability. It is of note that QAlb elevation was observed also in a substantial number of samples obtained during remission (median 213 days from last attack), demonstrating long-lasting BCB damage in MOG-EM (as previously seen also in AQP4-IgG-positive NMOSD [46]). It is unclear whether this reflects slow recovery from severe damage or rather ongoing subclinical inflammation, as suggested by the fact that MOG-IgG (just like AQP4-IgG [62]), remains detectable, partly at high levels, in many patients with MOG-EM also during remission.

l-lactate CSF levels were elevated in more than a quarter of our patients and, importantly, almost exclusively during acute attacks, rendering CSF l-lactate a possible marker of disease activity in MOG-EM (Table 6 and Fig. 2). This is similar to AQP4-IgG-positive NMOSD [46] but in contrast to MS, in which l-lactate levels are usually normal [63]. CSF l-lactate levels were particularly high in patients with acute myelitis and were strongly correlated with the cumulative spinal cord lesion load at the time of acute myelitis (Figs. 1 and 4). Surprisingly, CSF l-lactate levels also correlated significantly with QAlb. However, in contrast to albumin and TP, median l-lactate levels are physiologically higher in the CSF than in the serum. This renders it unlikely that the observed increase in CSF l-lactate levels was simply due to QAlb elevation and—in consequence—that the correlation of CSF l-Lactate with disease activity and lesion load was simply a result of increased BCB permeability [64, 65]. It thus seems more likely that CSF l-lactate and QAlb independently reflect the extent of intrathecal inflammation. CSF l-lactate levels were also strongly correlated with the CSF WCC.

Granulocytes are a known source of CSF l-lactate [66–70]. However, the frequency of l-lactate elevation did not differ between samples with and without granulocytes. Moreover, no significant correlation between CSF granulocyte counts and CSF l-lactate levels could be demonstrated in the present cohort. As a limitation, the number of samples with exact data on CSF granulocyte numbers was relatively small. l-lactate is thought to be produced also by astrocytes following glutamate stimulation [71, 72]. In NMOSD, in which we could also demonstrate a correlation between CSF l-lactate levels and the spinal cord lesion load, AQP4-IgG has been reported to result in increased extracellular glutamate concentrations due to coupled endocytosis of AQP4 and the excitatory amino acid transporter 2 (EAAT2) [73]. As previously discussed [74], an increase in extracellular glutamate could exert potentially detrimental effects also by overstimulating glutamate receptors in neurons and MOG-expressing oligodendrocytes [73]. It also renders oligodendrocytes susceptible to immunoglobulin-independent (alternative pathway) complement attack [73, 75]. However, there is no evidence so far for marked astrocytic dysfunction (e.g., resulting from inflammatory bystander damage) in MOG-EM, and extracellular glutamate concentrations have not been studied in MOG-IgG-positive patients to the best of our knowledge. Finally, neurons may switch to glycolysis, in particular if their capacity to metabolize anaerobically the lactate of astrocytic origin is exhausted [72]. Further studies are needed to better characterize the sources of intrathecal l-lactate in MOG-EM.

An elevated WCC was found in about 60% of samples from patients with active disease at the time of LP. Among CSF white cells, lymphocytes and monocytes were predominant, followed by neutrophils, an immune cell type never observed in MS (but in around 50% of samples from patients with acute attacks of AQP4-IgG-positive NMOSD [46]). In line with our demonstration of a lack of intrathecal MOG-IgG production in MOG-EM in a previous study [2], the lack of OCB and the normal QIgG values in most patients, and the lack of a positive MRZ reaction in our patients, antibody-secreting plasma cells were present only in 3.9% of all samples. The proportion of samples with activated lymphocytes (15.6%) was similar to that in AQP4-IgG-positive NMOSD (20.5%) but much lower than that usually seen in MS (> 75%) [33].

While a CSF WCC > 50 cells/μl is rare in MS and should prompt physicians to challenge the diagnosis, white cell numbers > 50 were observed in 19% of all samples, in 27% of all samples with pleocytosis, and in as many as 46% of those taken during acute myelitis (52% if lesions were longitudinally extensive). In the acute MY subgroup, WCC exceed even 100 cells/μl in every third patient; such high white cell numbers are virtually never seen in MS.

Neutrophil granulocytes or elevated CSF lactate levels, two laboratory features of bacterial CNS infection, were frequently observed during acute attacks. Granulocytes are also detectable in the CSF during very early-stage viral encephalomyelitis. Given the fact that MOG-EM attacks (in common with NMOSD attacks [15, 56]) are often preceded by infections [3, 4] which may result in fever or blood leukocytosis, this might well lead to the false suspicion of infectious disease in some cases. However, in most samples, both CSF lactate levels and absolute CSF white cell numbers were much lower in MOG-EM than in typical bacterial meningitis. While lactate concentrations exceeded the age-dependent reference range in 26% of all cases, lactate concentrations > 3 or > 4 mmol/l, as seen in a majority of patients with acute bacterial meningitis, were absent in 90% and 98% of cases, respectively.

Eosinophilic infiltration is not a typical feature of MOG-EM [76, 77]. In line with that observation, eosinophils were absent in all but 2 samples in the present cohort (1 × 1% of all white cells; 1 × number not specified). This is similar to MS, in which eosinophils are typically absent in the CSF, too. By contrast, previous studies have demonstrated the presence of eosinophil attractants in the CSF of patients with NMO [78], eosinophilic infiltration in NMO lesions [61], and the presence of eosinophils in 10-15% of acute CSF samples from patients with AQP4-IgG-positive NMOSD [46].

It is important to keep in mind that CSF pathology in MOG-EM varies markedly depending on disease activity, attack severity, and lesion location, and may be even absent in some cases. Of note, CSF pathology was strikingly less severe and less frequent in samples obtained during acute attacks of ON than in acute myelitis (Fig. 1). Patients presenting with isolated brain lesions exhibited CSF alterations more severe than in ON but less severe than in myelitis. These findings are well in line with the fact that the lesion volume is rather small in ON compared with myelitis (median lesion load 5 VS; up to 21 VS; LETM in 64%). Moreover, lumbar CSF in general does not reflect supratentorial lesions well due to its remoteness from the actual site of inflammation (so-called caudal–rostral CSF gradient). Moreover, we found highly significant differences in terms of CSF pathology (especially with regard to WCC, pleocytosis rate, QAlb, and TP) between attacks classified as “severe” by the treating physicians and attacks classified as only “mild” or “moderate” in this study. Future studies should attempt to define more objective measures for attack severity classification.

With the re-integration of OCB in the latest revision of the diagnostic criteria for MS [79] and the demonstration of substantial differences in CSF profiles between MS and its most important mimics [36, 38, 46, 57, 80–82], LP may be performed more often in the future. Although LP is a relatively safe procedure and routinely used in many countries, adverse event such as headache (post-puncture CSF pressure syndrome, the frequency of which can be substantially lowered by use of so-called atraumatic 22-24 gauge needles with conical tip and lateral opening [“Sprotte needles”]), radicular symptoms, non-specific back pain, disc prolapse, or aseptic disc necrosis (extremely rare), bleeding or infection rarely occur and a number of absolute (increased intracranial pressure with progressive herniation as indicated clinically and/or by MRI or CT; inflammatory infiltration of the skin in the puncture area) and relative (platelet counts < 50 GPt/L; therapeutic heparinization; oral anticoagulation) contraindications exist [33]. In consequence, patients should be thoroughly examined and contraindications carefully considered before performing LP. For a more detailed review of LP techniques and the prevention and management of complications see [33, 34].

### Strengths and limitations

We count among the particular strengths of this study the high number of patients included (given the rarity of the disease), the large number of both samples and parameters analyzed, the stratified analysis taking into account the clinical presentation at the time of LP, and, in particular, the fact that, in contrast with most previous studies on MOG-EM, samples were tested for MOG-IgG by means of at least two methodologically independent assays in almost all cases.

It is a potential limitation that our study was performed retrospectively and included a large number of university centers. However, the rarity of MOG-IgG-positive EM means that prospective monocenter studies cannot be performed if sufficient sample numbers are to be analyzed. Moreover, the multicenter approach reduces the risk of selection bias. Second, MRI data were obtained retrospectively. Although the correlation of lactate and TP levels with the spinal cord lesion load found in our cohort is intriguing, further studies are needed to confirm this finding in a prospective fashion. Third, no statistically significant differences were observed between treated and untreated patients in this cohort; however, as a limitation, it must be kept in mind that the number of samples obtained during immunosuppressive treatment at the time of LP was low. Accordingly, a type-II error cannot be excluded. As a general rule, LP should be performed before commencement of immunosuppressive or immunomodulatory treatment in patients with suspected inflammatory CNS disorders if the procedure is considered diagnostically relevant and not expected to delay treatment significantly.

## Conclusion

In summary, our study, the first to review comprehensively and systematically the CSF findings in MOG-EM in a large cohort of patients of mainly Caucasian descent, demonstrates that (i.) in sharp contrast to classic MS, intrathecal IgG synthesis is rare in MOG-IgG-positive EM, as shown both qualitatively and quantitatively; and (ii.), if present, intrathecal IgG synthesis is low in most patients, often transient, and mainly restricted to acute attacks (again in contrast to MS). Moreover, our data show that (iii.) CSF findings in acute myelitis differ substantially and significantly from those in acute ON (normal CSF findings are frequent in ON and do not exclude the diagnosis), which is of high potential clinical relevance; that (iv.) CSF findings in “monophasic” MOG-EM are not significantly different from those in relapsing MOG-EM; and that, different from MS, (v.) the degree of CSF alteration depends significantly on disease activity and attack severity (and could thus have potential prognostic value) in MOG-IgG-positive patients. Notably, (vi.) CSF L-lactate levels, QAlb, and CSF TP levels correlated with the spinal cord lesion load in patients with acute myelitis (again suggesting a potential prognostic value of LP in MOG-EM). Our finding that (vii.) CSF white cell numbers in MOG-EM may well exceed those typically observed in MS, in particular in acute myelitis (> 50 cells/μl in around 50% during acute LETM); that (viii.) a lack of pleocytosis, on the other hand, does not rule out the condition but is a frequent finding (around 66% in acute ON), and that (ix.) the intrathecal, polyclonal antiviral immune response (so-called MRZ reaction) discriminates sharply between MOG-EM and MS, and a positive MRZ reaction in patients with suspected MOG-EM may indicate a false-positive MOG-IgG result are all of differential diagnostic relevance. Moreover, (x.) neutrophilic pleocytosis and elevated l-lactate CSF concentrations render the condition—just like AQP4-IgG-positive NMOSD—a relevant differential laboratory diagnosis of (especially nonpurulent or chronic) bacterial infection in a subset of patients. Finally, we show that (xi.) QAlb and WCC are relatively frequently elevated also during remission, indicating sustained blood CSF barrier dysfunction and possibly subclinical inflammation in patients with MOG-EM. In many respects, CSF findings in MOG-EM share much more similarities with NMOSD than with MS. Our data may help to improve the differential diagnosis of MOG-EM and MS and to extend our understanding of the immunopathology of this newly described entity. A detailed analysis of the CSF findings in pediatric patients with MOG-EM can be found in part 2 of this article series [83].

## Supplementary information

**Additional file 1: Supplementary Table 1.** CSF findings during acute attacks and during remission in the ‘acute MY subgroup’, the ‘acute ON subgroup’ and the ‘acute BRAIN subgroup’ (stratified results from Table 11).

**Additional file 2: Supplementary Table 2.** Variations in OCB positivity over time.

**Additional file 3: Supplementary Table 3.** CSF findings in MOG-IgG-positive acute longitudinally extensive transverse myelitis (LETM) and MOG-IgG-positive non-longitudinally extensive transverse myelitis (NETM).

**Additional file 4: Supplementary Table 4.** CSF findings in MOG-IgG-positive acute bilateral ON and in MOG-IgG-positive acute unilateral ON.

**Additional file 5: Supplementary Figure 1.** CSF white cell counts in the ‘acute MY subgroup’, the ‘acute BRAIN subgroup and the ‘acute ON subgroup’.

**Additional file 6: Supplementary Figure 2.** No statistically significant differences in serum IgG, IgM, IgA and albumin levels between the ‘acute MY’, the ‘acute ON’ and the ‘acute BRAIN’ (B) subgroup.

**Additional file 7: Supplementary Figure 3.** Regression analysis of QAlb and CSF total protein demonstrated a close relationship between the two parameters (r^2^=0.75, *p*<0.00001).

## Data Availability

The datasets generated and/or analyzed during the current study are not publicly available but can be obtained from the corresponding author upon reasonable request.

## Abbreviations

ADEM: Acute disseminated EM
AQP4: Aquaporin-4
AQP4-IgG: Aquaporin-4 immunoglobulin G
BCB: Blood-CSF barrier
CBA: Cell based assay
CSF: Cerebrospinal fluid
IF: Intrathecal fraction
IgG/M/A: Immunoglobulin G/M/A
IHC: Immunohistochemistry
IS: Intrathecal synthesis
LETM: Longitudinally extensive transverse myelitis
LP: Lumbar puncture
MOG: Myelin oligodendrocyte glycoprotein
MOGAD: MOG antibody-associated disease
EM: Encephalomyelitis
NMO: Neuromyelitis optica
NMOSD: NMO spectrum disorders
OCB: Oligoclonal bands
ON: Optic neuritis
QAlb: Albumin CSF/serum ratio
QIgG: IgG CSF/serum ratio
QIgM: IgM CSF/serum ratio
QIgA: IgA CSF/serum ratio
rON: Recurrent optic neuritis
