# Supplementary material for: Cerebrospinal fluid findings in patients with myelin oligodendrocyte glycoprotein (MOG) antibodies. Part 1: Results from 163 lumbar punctures in 100 adult patients
Source: J Neuroinflammation. 2020 Sep 3;17:261. doi: 10.1186/s12974-020-01824-2 (PMC7470615; doi:10.1186/s12974-020-01824-2)
Supplement: Supplementary file 1 — Additional file 1: Supplementary Table 1. CSF findings during acute attacks and during remission in the ‘acute MY subgroup’, the ‘acute ON subgroup’ and the ‘acute BRAIN subgroup’ (stratified results from Table 11). [file 12974_2020_1824_MOESM1_ESM.pdf]

	Units	Attack MY, first LP/event	Remission MY, last LP/event	Attack ON, first LP/event	Remission ON, last LP/event	Attack BRAIN, first LP/event	Remission BRAIN, last LP/event
Pleocytosis	*samples*	32/40 (80%)	1/6 (16.7%)	15/50 (30%)	0/6 (0%)	6/10 (60%)	1/3 (33.3%)
WCC	*cells/µl*	31.5 (1-463;40)	1.5 (0-13;6)	3 (0-135;48)	1 (0-3;5)	13.5 (0-68;10)	3 (1-16;3)
WCC >100/µl	*samples*	12/40 (30%)	0/6 (0%)	1/49 (2%)	0/6 (0%)	0/10 (0%)	0/3 (0%)
OCB	*samples*	3/37 (8.1%)	1/6 (16.7%)	3/48 (6.3%)	0/6 (0%)	2/10 (20%)	0/3 (0%)
IgG-IF >10%	*samples*	2/35 (5.7%)	0/6 (0%)	2/40 (5%)	0/4 (0%)	0/8 (0%)	0/3 (0%)
QAlb > Qlim(Alb)	*samples*	22/36 (61.1%)	2/6 (33.3%)	15/43 (34.9%)	1/4 (25%)	4/8 (50%)	0/3 (0%)
CSF TP elevated	*samples*	21/37 (56.8%)	1/6 (16.7%)	15/47 (31.9%)	1/6 (16.7%)	6/8 (75%)	1/3 (33.3%)
CSF TP concentrations	*mg/dl*	59.5 (20.5-176;34)	31.05 (5.11-48.2;6)	37.4 (20.8-171.8;43)	37.85 (32-59.8;4)	48.2 (30.4-74.2;8)	40 (20-47;3)
CSF TP >100 mg/dl	*samples*	6/37 (16.2%)	0/6 (0%)	1/47 (2.1%)	0/6 (0%)	0/8 (0%)	0/3 (0%)
CSF L-lactate elevated	*samples*	10/26 (38.5%)	0/4 (0%)	6/36 (16.7%)	0/4 (0%)	0/3 (0%)	0/2 (0%)
CSF L-lactate concentrations	*mg/dl*	2.14 (1.12-4.43;24)	1.45 (1.4-1.9;4)	1.7 (1.05-2.68;35)	1.7 (1.44-1.74;3)	1.51 (1.5-1.7;3)	1.51 (1.33-1.69;2)
CSF L-lactate >3 mmol/l	*samples*	4/26 (15.4%)	0/4 (0%)	0/36 (0%)	0/4 (0%)	0/3 (0%)	0/2 (0%)
Time since attack onset	*days*	7 (0-31;41)	380.5 (119-3664;6)	8.5 (0-34;50)	154 (97-275;6)	14 (0-24;11)	191 (117-2624;3)

Supplementary Table 1. C SF findings during acute attacks and during remission in the 'acute MY subgroup', the 'acute ON subgroup' and the 'acute BRAIN subgroup' (stratified results from Table 11). BRAIN = brain/brainstem/cerebellar subgroup; CSF = cerebrospinal fluid; IgG-IF = intrathecally produced CSF immunoglobulin G fraction; LP = lumbar puncture; MY = myelitis subgroup; ON = optic neuritis subgroup; QAlb = albumin CSF/serum ratio; TP = total protein; WCC = white cell count.
