# Supplementary material for: Cerebrospinal fluid findings in patients with myelin oligodendrocyte glycoprotein (MOG) antibodies. Part 1: Results from 163 lumbar punctures in 100 adult patients
Source: J Neuroinflammation. 2020 Sep 3;17:261. doi: 10.1186/s12974-020-01824-2 (PMC7470615; doi:10.1186/s12974-020-01824-2)
Supplement: Supplementary file 2 — Additional file 2: Supplementary Table 2. Variations in OCB positivity over time. [file 12974_2020_1824_MOESM2_ESM.pdf]

	LP #1	LP #2	LP #3	LP #4	LP #5
Pat. #1	OCB –/1, M, Rem, QIgG neg	OCB –/1, M, Rem, QIgG neg	OCB –/4, M, Rem, QIgG neg	OCB –/4, M, Rem, QIgG neg	**OCB +/2,** M, Rem, QIgG neg
Pat. #2	**OCB +/2,** M, A, QIgG neg	OCB –/1, M, A, QIgG neg			
Pat. #3	OCB –/1, ON, A, QIgG neg	**OCB +/2,** nd, A?, **QIgG pos**			
Pat. #4	OCB –/1, ON, A, QIgG n.d.	OCB –/4, ON, Rem, QIgG neg			
Pat. #5	OCB –/1, nd, Rem, QIgG neg	OCB –/4, M, A, QIgG nd	OCB –/4, M, A, QIgG nd	OCB –/4, M, A, QIgG nd	OCB –/1, ON, A, QIgG nd
Pat. #6	**OCB +/3,** M+B, A, **QIgG pos**	**OCB +/3,** M+B, A, QIgG neg	**OCB +/2,** M+B, A, **QIgG pos**		
Pat. #7	OCB –/1, ON, A, QIgG neg	OCB –/4, M, A, QIgG neg			
Pat. #8	OCB –/1, ON, A, QIgG neg	OCB –/4, ON, A, QIgG neg			
Pat. #9	OCB –/4, ON, A, QIgG nd	OCB –/1, B, A, QIgG neg			

Supplementary Table 2. Variations in OCB positivity over time. In 1 patient, OCB disappeared over the course of disease (absent one week after the initial LP and following steroid treatment); in 2 patients, OCB were absent in the initial sample but were detectable in a follow-up sample taken 271 and 66 days later, respectively. Most samples were obtained during acute disease with the only exception of patient #1, in whom LP was performed five times during remission. Note that QIgG was normal in most of the OCB-positive samples, indicating low levels of intrathecal IgG synthesis. A = acute attack; B = brain/brainstem/cerebellar disease (last attack before LP); M = myelitis (last attack before LP); OCB = oligoclonal bands; ON = optic neuritis (last attack before LP); QIgG = CSF/serum immunoglobulin G ratio; Rem = remission.
