# Supplementary material for: Cerebrospinal fluid findings in patients with myelin oligodendrocyte glycoprotein (MOG) antibodies. Part 1: Results from 163 lumbar punctures in 100 adult patients
Source: J Neuroinflammation. 2020 Sep 3;17:261. doi: 10.1186/s12974-020-01824-2 (PMC7470615; doi:10.1186/s12974-020-01824-2)
Supplement: Supplementary file 3 — Additional file 3: Supplementary Table 3. CSF findings in MOG-IgG-positive acute longitudinally extensive transverse myelitis (LETM) and MOG-IgG-positive non-longitudinally extensive transverse myelitis (NETM). [file 12974_2020_1824_MOESM3_ESM.pdf]

	Units	Acute LETM, first LP/event	Acute NETM, first LP/event
Pleocytosis	*samples*	20/24 (83.3%)	11/15 (73.3%)
WCC	*cells/µl*	45 (1-463;24)	29 (1-234;15)
WCC >100/µl*	*samples*	9/24 (37.5%)	3/15 (20%)
OCB	*samples*	2/23 (8.7%)	1/13 (7.7%)
IgG-IF >10%	*samples*	1/21 (4.8%)	1/14 (7.1%)
QAlb > Qlim(Alb)	*samples*	14/22 (63.6%)	8/14 (57.1%)
CSF TP elevated	*samples*	13/22 (59.1%)	8/14 (57.1%)
CSF TP concentrations	*mg/dl*	67.4 (20.5-176;20)	53 (23-101;14)
CSF L-lactate elevated§	*samples*	8/15 (53.3%)	2/10 (20%)
CSF L-lactate concentrations#	*mg/dl*	2.2 (1.3-4.43;14)	1.8 (1.12-2.6;10)
Time since attack onset	*days*	5 (0-31;25)	10 (2-29;15)

Supplementary Table 3. CSF findings in MOG-IgG-positive acute longitudinally extensive transverse myelitis (LETM) and MOG-IgG-positive non-longitudinally extensive transverse myelitis (NETM). Note that only the first LP obtained during an acute event was considered for this analysis to control for the fact that the number of CSF samples obtained per event differed among patients. Values represent rates (with percentages given in brackets) or medians (with ranges and patient numbers given in brackets), respectively. *p=n.s.; §p=n.s.; #p<0.05. CSF = cerebrospinal fluid; IgG-IF = intrathecally produced CSF immunoglobulin G fraction; LP = lumbar puncture; OCB = oligoclonal bands; QAlb = CSF/serum albumin ratio; TP = total protein; WCC = white cell count.
