# Supplementary material for: Cerebrospinal fluid findings in patients with myelin oligodendrocyte glycoprotein (MOG) antibodies. Part 1: Results from 163 lumbar punctures in 100 adult patients
Source: J Neuroinflammation. 2020 Sep 3;17:261. doi: 10.1186/s12974-020-01824-2 (PMC7470615; doi:10.1186/s12974-020-01824-2)
Supplement: Supplementary file 4 — Additional file 4: Supplementary Table 4. CSF findings in MOG-IgG-positive acute bilateral ON and in MOG-IgG-positive acute unilateral ON. [file 12974_2020_1824_MOESM4_ESM.pdf]

	Units	Acute bilateral ON, first LP/event	Acute unilateral ON, first LP/event
Pleocytosis*	*samples*	4/10 (40%)	10/37 (27%)
WCC	*cells/µl*	5 (0-135;3)	3 (0-29;5)
WCC >100	*samples*	1/10 (10%)	0/36 (0%)
OCB	*samples*	0/10 (0%)	2/35 (5.7%)
IgG-IF >10%	*samples*	0/9 (0%)	1/28 (3.6%)
QAlb > Qlim(Alb)	*samples*	5/9 (55.6%)	10/31 (32.3%)
CSF TP elevated	*samples*	4/9 (44.4%)	10/35 (28.6%)
CSF TP concentrations	*mg/dl*	41.4 (25.77-92.9;10)	37 (20.8-171.8;34)
CSF L-lactate elevated	*samples*	2/8 (25%)	4/25 (16%)
CSF L-lactate concentrations	*mg/dl*	1.59 (1.4-2.26;8)	1.75 (1.05-2.68;26)
Time since attack onset	*days*	10 (2-18;13)	7 (0-34;42)

Supplementary Table 4. CSF findings in MOG-IgG-positive acute bilateral ON and in MOG-IgG-positive acute unilateral ON. Note that only the first LP obtained during each acute event was taken into account for this analysis to control for the fact that the number of CSF samples obtained per event differed among patients. *p=n.s. CSF = cerebrospinal fluid; IgG-IF = intrathecally produced CSF immunoglobulin G fraction; OCB = oligoclonal bands; QAlb = CSF/serum albumin ratio; TP = total protein; WCC = white cell count.
