# Supplementary material for: Cerebrospinal fluid findings in patients with myelin oligodendrocyte glycoprotein (MOG) antibodies. Part 1: Results from 163 lumbar punctures in 100 adult patients
Source: J Neuroinflammation. 2020 Sep 3;17:261. doi: 10.1186/s12974-020-01824-2 (PMC7470615; doi:10.1186/s12974-020-01824-2)
Supplement: Supplementary file 5 — Additional file 5: Supplementary Figure 1. CSF white cell counts in the ‘acute MY subgroup’, the ‘acute BRAIN subgroup and the ‘acute ON subgroup’. [file 12974_2020_1824_MOESM5_ESM.pdf]

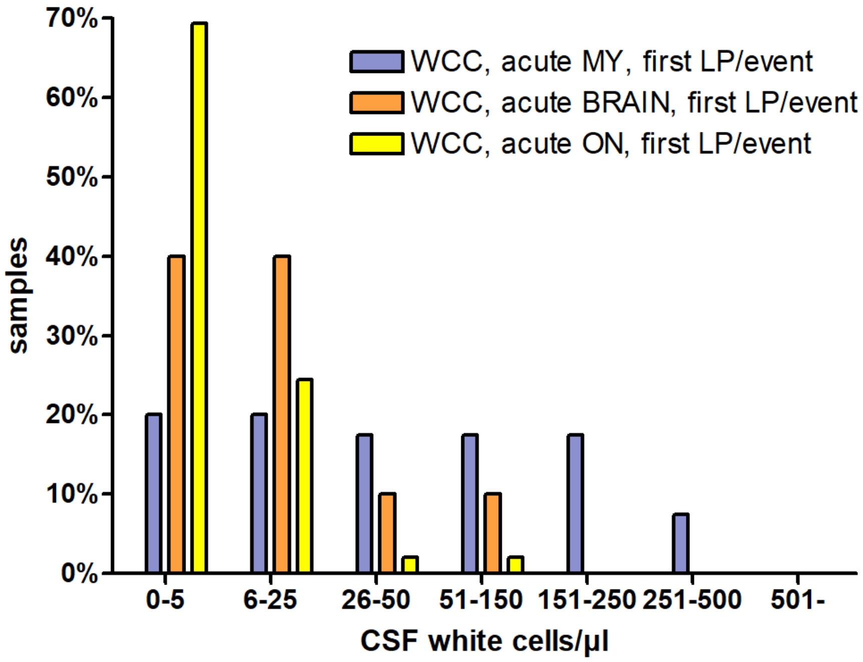

Supplementary Figure 1. CSF white cell counts in the 'acute MY subgroup', the 'acute BRAIN subgroup' and the 'acute ON subgroup'.
