# Supplementary material for: Cerebrospinal fluid findings in patients with myelin oligodendrocyte glycoprotein (MOG) antibodies. Part 1: Results from 163 lumbar punctures in 100 adult patients
Source: J Neuroinflammation. 2020 Sep 3;17:261. doi: 10.1186/s12974-020-01824-2 (PMC7470615; doi:10.1186/s12974-020-01824-2)
Supplement: Supplementary file 6 — Additional file 6: Supplementary Figure 2. No statistically significant differences in serum IgG, IgM, IgA and albumin levels between the ‘acute MY’, the ‘acute ON’ and the ‘acute BRAIN’ (B) subgroup. [file 12974_2020_1824_MOESM6_ESM.pdf]

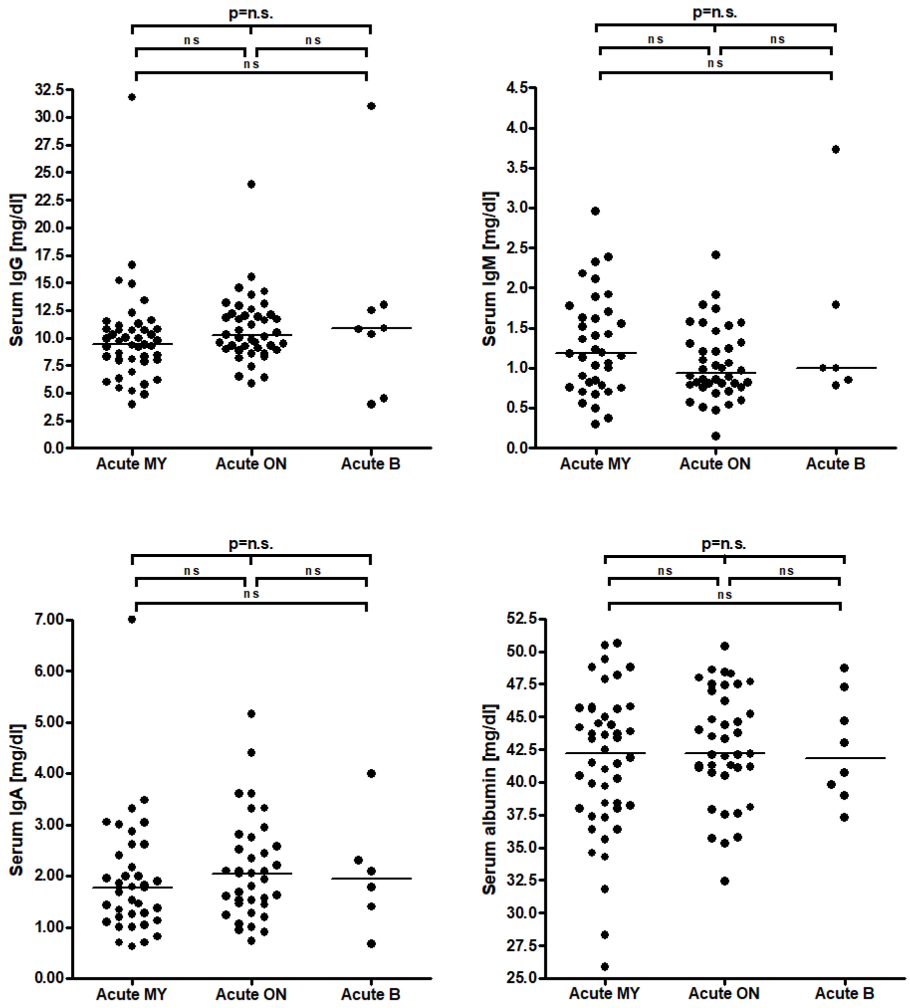

Supplementary Figure 2. No statistically significant differences in serum IgG, IgM, IgA and albumin levels between the 'acute MY', the 'acute ON' and the 'acute BRAIN' subgroup. B = brain; CSF = cerebrospinal fluid; MY = myelitis; ON = optic neuritis.
