# Supplementary material for: Cerebrospinal fluid findings in patients with myelin oligodendrocyte glycoprotein (MOG) antibodies. Part 1: Results from 163 lumbar punctures in 100 adult patients
Source: J Neuroinflammation. 2020 Sep 3;17:261. doi: 10.1186/s12974-020-01824-2 (PMC7470615; doi:10.1186/s12974-020-01824-2)
Supplement: Supplementary file 7 — Additional file 7: Supplementary Figure 3. Regression analysis of QAlb and CSF total protein demonstrated a close relationship between the two parameters (r2=0.75, p<0.00001). [file 12974_2020_1824_MOESM7_ESM.pdf]

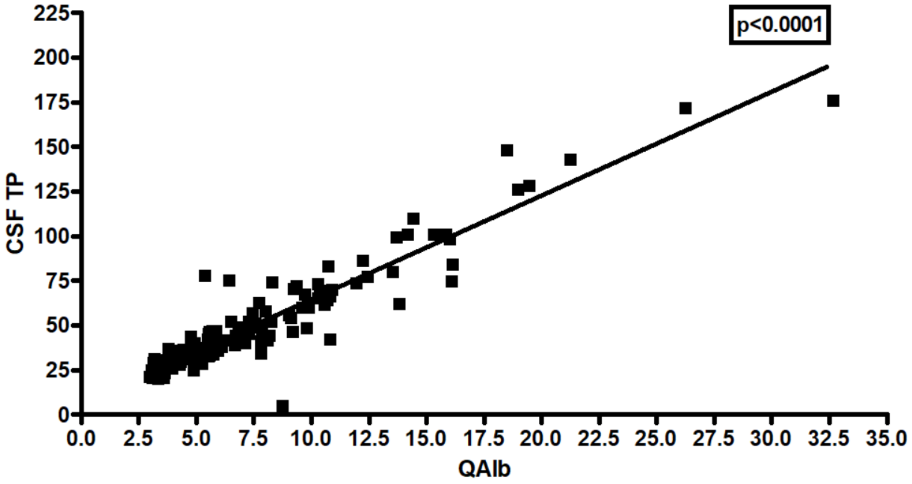

Supplementary Figure 3. Regression analysis of QAlb and CSF total protein, demonstrating a close relationship between the two parameters (r^2=0.874, p<0.00001). CSF = cerebrospinal fluid; QAlb = albumin CSF/serum ratio; TP = total protein.
